# Promotion of Anti-Tuberculosis Macrophage Activity by L-Arginine in the Absence of Nitric Oxide

**DOI:** 10.3389/fimmu.2021.653571

**Published:** 2021-05-14

**Authors:** Melanie C. McKell, Rebecca R. Crowther, Stephanie M. Schmidt, Michelle C. Robillard, Rachel Cantrell, Maria A. Lehn, Edith M. Janssen, Joseph E. Qualls

**Affiliations:** ^1^ Department of Pediatrics, University of Cincinnati College of Medicine, Cincinnati, OH, United States; ^2^ Division of Infectious Diseases, Cincinnati Children’s Hospital Medical Center, Cincinnati, OH, United States; ^3^ Immunology Graduate Program, University of Cincinnati College of Medicine, Cincinnati, OH, United States; ^4^ Medical Scientist Training Program, University of Cincinnati College of Medicine, Cincinnati, OH, United States; ^5^ Division of Immunobiology, Cincinnati Children’s Hospital Medical Center, Cincinnati, OH, United States

**Keywords:** macrophage, arginine, citrulline, tuberculosis, glycolysis

## Abstract

Macrophages are indispensable immune cells tasked at eliminating intracellular pathogens. *Mycobacterium tuberculosis* (*Mtb*), one of the most virulent intracellular bacterial pathogens known to man, infects and resides within macrophages. While macrophages can be provoked by extracellular stimuli to inhibit and kill *Mtb* bacilli, these host defense mechanisms can be blocked by limiting nutritional metabolites, such as amino acids. The amino acid L-arginine has been well described to enhance immune function, especially in the context of driving macrophage nitric oxide (NO) production in mice. In this study, we aimed to establish the necessity of L-arginine on anti-*Mtb* macrophage function *independent* of NO. Utilizing an *in vitro* system, we identified that macrophages relied on NO for only half of their L-arginine-mediated host defenses and this L-arginine-mediated defense in the absence of NO was associated with enhanced macrophage numbers and viability. Additionally, we observed macrophage glycolysis to be driven by both L-arginine and mechanistic target of rapamycin (mTOR), and inhibition of glycolysis or mTOR reduced macrophage control of *Mtb* as well as macrophage number and viability in the presence of L-arginine. Our data underscore L-arginine as an essential nutrient for macrophage function, not only by fueling anti-mycobacterial NO production, but also as a central regulator of macrophage metabolism and additional host defense mechanisms.

## Introduction

*Mycobacterium tuberculosis* (*Mtb*), the causative agent of tuberculosis, is a persistent pathogen which continues to plague a quarter of the world’s population despite the availability of antibiotics (1, 2). Integral to the host-pathogen interaction, macrophages are the main cellular niche for *Mtb*, have the ability to kill *Mtb* with appropriate stimuli from peripheral immune cells, and can initiate granuloma formation following infection (3–6). The extracellular environment encountered prior to and throughout infection can have substantial effects on the macrophage response to infection. Peripheral immune cells, especially T cells and NK cells that produce interferon (IFN)-γ, are necessary to enhance macrophage activation (4, 7–10). This increased activation includes amplified killing mechanisms, such as production of nitric oxide (NO), phagosomal maturation, and increased antigen presentation and co-stimulatory molecule expression (6). The macrophage inflammatory state is not only influenced by cytokines, but also by nutrients. For example, modulating availability of carbohydrates, lipids, amino acids, and vitamins can have substantial effects on macrophage inflammatory vigor (11–13). A metabolite of particular interest is the amino acid L-arginine.

L-arginine is a central metabolite for multiple cellular processes in macrophages and other cells. Aside from protein synthesis, downstream metabolites of L-arginine include creatine, agmatine, L-citrulline, and L-ornithine, which can contribute to cell proliferation (polyamine synthesis) and wound healing (proline synthesis) (14). L-arginine also has many immunomodulatory functions, the most prominent in mouse macrophages being the conversion of L-arginine into NO and L-citrulline *via* the enzyme inducible NO synthase (iNOS). NO is a potent microbicidal agent, critical for control of *Mtb* in mouse macrophages (15–17). When extracellular L-arginine is limiting, enzymatic “recycling” of L-citrulline to L-arginine occurs in mouse macrophages to maintain maximal NO production (18–20). Indeed, our previous work has shown that L-arginine synthesis is necessary for optimal host defense against *Mtb in vivo* (19, 21).

Apart from the contribution of NO production to host defense, L-arginine drives effector functions of many immune cells. For example, T cells rely on L-arginine for proliferation and cytokine production, NK cells require L-arginine for activation and cytotoxicity, and neutrophils release the L-arginine-sequestering enzyme arginase into the extracellular environment to limit L-arginine available to other cells, effectively suppressing L-arginine-dependent immune responses (22–24). Considering the array of effector functions regulated by L-arginine, we hypothesized that L-arginine coordinates additional macrophage anti-mycobacterial defenses that are not reliant on NO production.

In this study, we employed cell culture techniques allowing us to determine the contribution of L-arginine – or the L-arginine precursor, L-citrulline – on anti-mycobacterial macrophage activity in the absence of NO. L-arginine was found to support NO-independent macrophage anti-mycobacterial activity, which was associated with enhanced glycolysis. Inhibiting glycolysis or an upstream regulator of glycolysis – mechanistic target of rapamycin (mTOR) – blocked control of *Mtb* by macrophages in the absence of NO. Additionally, we found L-arginine to support macrophage number and viability, which were associated with optimal mycobacterial control. Finally, exogenous NO could only partially restore *Mtb* inhibition in the absence of L-arginine as compared to macrophages cultured in L-arginine. Collectively, these data indicate the importance of adequate nutrient availability – especially that of L-arginine – for macrophage-mediated control of *Mtb.*

## Materials and Methods

### Mice

All mice were housed and bred within the Division of Veterinary Services at Cincinnati Children’s Hospital Medical Center (CCHMC). Strains were originally obtained from The Jackson Laboratories: C57BL/6J, 000664; B6.Cg-Tg(Tek-cre)1Ywa/J, 008863; B6.129S7-*Asl* ^tm1Brle^/J, 018830; B6.129P2-*Nos2^tm1Lau^*/J, 002609). *Ass1* ^flox/flox^;Tie2-cre mice were originally a gift from S. E. Kohler (25). Argininosuccinate lyase (Asl) and argininosuccinate synthase (Ass1) mutant mice were bred to Tie2-cre mice to generate macrophages deficient in these enzymes (noted as *Asl* ^-/-^ and *Ass1* ^-/-^ herein). All mice are on the C57Bl/6 background (wild type, [WT]). All procedures were approved by the Institutional Animal Care and Use Committee at CCHMC in accordance with the Guide for the Care and Use of Laboratory Animals.

### Cell Culture

#### Media

Complete Dulbecco’s Modified Eagles Media (C-DMEM) was prepared by adding bovine calf serum (SH30073.03, Thermo Fisher Scientific) to 10%, and penicillin/streptomycin (15140-122, Gibco, Life Technologies) to 1% in standard DMEM (10-013-CV, Cellgro, Corning Life Sciences). L-arginine-free (R-free) C-DMEM lacking phenol red was prepared by adding dialyzed fetal bovine serum (35-071-CV, Cellgro, Corning Life Sciences) to 10% in SILAC DMEM (D9443, Sigma-Aldrich) plus L-glutamine (584 mg/L, 25030-081, Life Technologies), L-lysine-HCl (146.2 mg/L, L8662, Sigma-Aldrich), L-leucine (52.5 mg/L, L8912, Sigma-Aldrich), sodium pyruvate (110 mg/L, 11360-070, Life Technologies), D-glucose (4500 mg/L, G8796, Sigma-Aldrich) and phenol red (15 mg/L p3532, Sigma-Aldrich). For luminescence experiments, phenol red was excluded from cell culture media. L-arginine (A8094, Sigma-Aldrich), L-citrulline (C7629, Sigma-Aldrich), and L-arginine · HCl (A5131, Sigma-Aldrich) were prepared at a stock concentration of 100 mM in sterile water. L-arginine and L-citrulline stocks were added to R-free C-DMEM to concentrations noted in the text. Complete Roswell Park Memorial Institute 1640, (C- RPMI) was prepared by adding dialyzed fetal bovine serum to 10% and penicillin/streptomycin to 1% in standard RPMI 1640 (10-040-CV, Corning Life Sciences).

### Macrophages

#### Peritoneal Macrophages (PMΦs)

Mice were administered 1 ml sterile thioglycolate (R064710, Remel, Fisher Scientific) by intraperitoneal injection. After 4 days, peritoneal cells were collected by lavage. Following red cell lysis, cells were plated in C-DMEM at 2x10^5^, 6x10^5^, or 1x10^6^ macrophages per well in 96-, 24-, or 12-well flat bottom tissue culture plates, respectively. For luminescence assays, macrophages were plated on white 96-well tissue culture plate (353296, Fisher Scientific) in 200 μL C-DMEM. C-DMEM and non-adherent cells were aspirated after overnight incubation, and fresh R-free C-DMEM was added with concentrations of L-arginine or L-citrulline noted in the text. Cells were infected and/or stimulated with IFN-γ (2 ng/ml, 14-8311-63, Invitrogen, Thermo Fischer Scientific – discontinued; PMC4034, Gibco), Pam3Cys (100 ng/ml, tlrl-pms, Invivogen), 1400w (100 μM, 214358-33-5, Cayman Chemical), 2-deoxyglucose (2-DG, D8375, Sigma-Aldrich), rapamycin (A8167, ApexBio), torin 1 (10997, Cayman Chemical) and/or dimethyl sulfoxide (DMSO, D8418, Sigma-Aldrich, used as vehicle control for rapamycin and torin) where noted. DETA-NONOate (82120, Cayman Chemical) was added 24 hours post infection (40 μM) and then at 48 hours post-infection (80 μM) for the “high” dose, and only at 48 hours post-infection (80 μM) for the “low” dose.

#### RAW 264.7 Macrophages

Frozen aliquots of RAW 264.7 (ATCC TIB-71) cells were thawed in 37°C water bath and cultured in C-RPMI. Media was removed after overnight culture and replaced with fresh C-RPMI. Cells were scraped, counted, and added to 96-well flat bottom plates for 5-bromo-2’-deoxyuridine (BrdU) incorporation controls as described herein.

### Mycobacterium tuberculosis

*Mtb* H_37_R_v_ [luciferase expressing, gift from L. Schlesinger (26)] was handled in a biosafety level 3 facility. *Mtb* H_37_R_a_ (25177, ATCC) bacilli were handled with biosafety level 2 precautions. Bacilli were cultured in 7H9 media (M0178, Sigma-Aldrich) plus OADC (R450605, Remel, Fisher Scientific) containing 0.05% Tween-80 (P4780, Sigma-Aldrich) at 37°C shaking ~75 r.p.m. *Mtb* concentration was estimated by measuring absorbance at 600 nm and adjusted to appropriate concentrations in sterile phosphate buffered saline (PBS). *Mtb* H_37_R_v_ was adjusted to provide ~2x10^5^ mycobacteria to macrophages (multiplicity of infection [MOI]~1) unless otherwise noted. In some experiments, *Mtb* growth was determined in titrating concentrations of sodium lactate (L7022, Sigma-Aldrich) or lactic acid (199257, Sigma-Aldrich). Between 0 to 144 hours post-infection, mycobacterial viability was determined by analyzing relative luminescence units (RLUs) using a DTX-880 Multimode plate reader and detection software (Beckman Coulter). When % RLUs were used for analysis, raw RLUs have been documented in **Supplemental Table 1**.

### Griess Assay

Nitric oxide production was determined by Griess assay to detect ${\text{NO}}_{2}^{-}$ in cell culture supernatants. Equal volumes of cell culture supernatant and Griess reagent (0.5 g sulfanilamide (S9251, Sigma-Aldrich), 0.05 g N-(1-Naphthyl)ethylenediamine dihydrochloride (33461, Sigma-Aldrich), and 1.17 mL phosphoric acid per 50 mL water) were mixed in a 96-well plate. Sodium nitrite (237213, Sigma-Aldrich) was used as a standard. Absorbance values were measured immediately at 492 nm using a DTX-880 Multimode plate reader and detection software (Beckman Coulter).

### Cell Culture Acidity by Media Color Quantification

Media color absorbance at 560 nm was determined using a Synergy HTX Multi-Mode Reader and Gen5 software (BioTek). Acidity values were plotted as the inverse of the absorbance values on a log_2_ scale. Images of cell culture media were taken using a Cytation 5 Cell Imaging Multi-Mode Reader and Gen5 software (BioTek).

### Lactate Quantification

PMΦs were cultured in R-free C-DMEM containing 400 μM L-arginine, 400 μM L-citrulline, or neither amino acid. Cells were infected/stimulated as noted in the text. Supernatants were collected and frozen at -80°C prior to quantification. Lactate quantification was performed using the Lactate Assay Kit (MAK064-1KT, Sigma-Aldrich) according to the manufacturer’s instructions.

### Cellular Glycolytic Metabolism

Glycolytic extracellular acidification rate (ECAR) from PMΦs was determined using Seahorse technology (Agilent). The Seahorse XFe96 sensor cartridge (102416-100, Agilent) was hydrated overnight with 200 μL Seahorse XF Calibrant Solution (100840-000, Agilent) by wrapping plate in parafilm and incubating 24 hours at 37°C. Following incubation, the cartridge was removed and gently lifted from the cartridge holder to dislodge bubbles. The cartridge was replaced into holder with calibrant solution prior to loading with injection solutions immediately before running. PMΦs were cultured at 2x10^5^ cells per well in a Seahorse XFe96 cell culture microplate (102416-100, Agilent) in C-DMEM. After overnight adherence, cells were washed with PBS and cultured in R-free C-DMEM containing 400 μM L-arginine, 400 μM L-citrulline, or neither amino acid and stimulated with IFN-γ, Pam3Cys, rapamycin, DMSO, and/or 1400w where noted in text. After 24 hour incubation, cells were washed with PBS and incubated in a non-CO_2,_ 37°C incubator with R-free Seahorse media supplemented with 400 μM L-arginine · HCl, 400 μM L-citrulline, or neither amino acid for 1 hour prior to running. Media: R-free Seahorse media was prepared by combining CaCl_2_ (499609, Sigma-Aldrich), Fe(NO_3_)_3_ · 9H_2_O (f8508, Sigma-Aldrich), MgSO_4_ (anhydrous) (m2643, Sigma-Aldrich), phenol red (p3532, Sigma-Aldrich), NaH_2_PO_4_ · H_2_O (s3139, Sigma-Aldrich), KCl (p9541, Sigma-Aldrich), NaCl (s3014, Sigma-Aldrich), L-cystine · 2HCl (c2526, Sigma-Aldrich), glycine (161-0724, Bio-Rad), L-histidine · HCl · H_2_O (h5659, Sigma-Aldrich), L-isoleucine (i2752, Sigma-Aldrich), L-leucine (l8912, Sigma-Aldrich), L-lysine · HCl (l8662, Sigma-Aldrich), L-methionine (m9625, Sigma-Aldrich), L-phenylalanine (p2126, Sigma-Aldrich), L-serine (s4500, Sigma-Aldrich), L-threonine (t8625, Sigma-Aldrich), L-tryptophan (t0254, Sigma-Aldrich), L-valine (v0500, Sigma-Aldrich), L-tyrosine · 2Na · 2H_2_O (res3156ta7, Sigma-Aldrich), folic acid (f7876, Sigma-Aldrich), riboflavin (phr1054, Sigma-Aldrich) D-Ca-pantothenate (21210, Sigma-Aldrich), choline chloride (c7017, Sigma-Aldrich), myo-inositol (i5125, Sigma-Aldrich), nicotinamide (47865u, Sigma-Aldrich), pyridoxine · HCl (47862, Sigma-Aldrich), and thiamine · HCl (47858, Sigma-Aldrich) to concentrations found in Agilent Seahorse XF base medium (103334-100, Agilent). Glutamax (2 mM, 35050061, Gibco, Life Technologies) and glucose (10 mM, g7021, Sigma-Aldrich) were supplemented to media. ECAR values were obtained every 5 minutes for a total of 60 minutes on a Seahorse XFe96 Analyzer (Agilent). Following 30 minutes of run, 2-DG was injected into the wells at a final concentration of 100 mM.

### Crystal Violet Assay

Macrophage numbers were determined by crystal violet assay, modified from (27). Briefly, cell culture media was removed and cells were gently washed with water. Cells were incubated with 0.05% crystal violet solution (20 ml 95% ethanol, 80 ml water, and 0.5 g crystal violet powder (C6158, Sigma-Aldrich)) for 20 minutes at room temperature. Crystal violet solution was removed and cells were gently washed with water and allowed to air dry. After fully drying, 100% methanol was used to saturate the remaining crystal violet solution for 20 minutes. Absorbance was read at 595 nm using a DTX-880 Mutltimode plate reader (Beckman Coulter) or at 570 nm using a Synergy HTX Multi-Mode Reader and Gen5 software (BioTek) and plotted against a standard curve of known macrophage numbers.

### AlamarBlue Cell Viability Assay

Following 72 hours in culture, 90 µL media was removed and 10 µL alamarBlue (DAL1025, Invitrogen) was added to all wells. Macrophages were incubated for an additional 2 hours at 37°C plus 5% CO_2_ prior to reading fluorescence according to the manufacturer’s protocol. Fluorescence values were obtained using a DTX-880 Mutltimode plate reader (Beckman Coulter) or Synergy Neo2 Multi-Mode Reader and Gen5 software (BioTek) and normalized to fluorescence obtained from wells without macrophages. Values are noted as the relative fluorescence units (RFUs).

### BrdU Incorporation Proliferation Assay

Macrophage proliferation was determined by BrdU incorporation (6813, Cell Signaling) according to the manufacturer’s protocol. BrdU was detected following 72 hours of incorporation (PMΦs) or 20 hours of incorporation (RAW 264.7). Absorbance values at 450 nm were obtained using a DTX-880 Multimode plate reader (Beckman Coulter) or Synergy Neo2 Multi-Mode Reader and Gen5 software (BioTek).

### RNA Analysis

Cell culture media was aspirated and macrophages were lysed in Trizol Reagent (15596018, Invitrogen, Fisher Scientific) and prepared for RNA purification following the manufacturer’s protocol. RNA was converted to cDNA using SuperScript II reverse transcriptase (18064-014, Invitrogen) with a mixture of oligo dT (18418012, Invitrogen) and random primers (48190-011, Invitrogen). cDNA was amplified using SybrGreen (4309155, Applied Biosystems) and analyzed by quantitative real time polymerase chain reaction (qRT-PCR) on a 7500 Fast Real-Time PCR System (Applied Biosystems). Primers: hexokinase 1 (Hk1, F: 5` GGTCTACGACACCCCAGAGA 3`, R: 5` TCCTTGATCTTCCTTTTCTCCA 3`; lactate dehydrogenase a (Ldha, F: 5` GACAAGGAGCAGTGGAAGGA 3`, R: 5` GCCCAGGATGTGTAACCTTT 3`); monocarboxylate transporter 4 (Mct4, F: 5` GAATGGTGGCTGCGTCCT 3`, R: 5` GATGAGGGAAGGCTGGAAGT 3`); RNA Polymerase 2 Subunit A (Polr2a, F: 5` AGGACACTGGACCGCTCATG 3`, R: 5` GCATAATATTCTCAGAGACTCCCTTCA 3`).

### Immunoblot

Following overnight adherence, PMΦs were washed with PBS and fresh R-free C-DMEM or R-free C-DMEM containing 400 μM L-arginine was added. Cells were infected/stimulated as noted in the text, followed by one PBS wash on ice, and lysing in RIPA. RIPA lysates were separated by Tris–HCl buffered 4–15% gradient SDS-PAGE (3450028, Bio-Rad), followed by transfer to Amersham Protran 0.2 μm nitrocellulose blotting membranes (10600006, GE Healthcare). Membranes were blocked in 3% milk (1706404, Bio-Rad) in TBS (10 ml 1M Tris base [T6066, Sigma] at pH 8.0 and 30 ml 5M NaCl [S3014, Sigma] QS with water to 1L) plus 10% Tween-20 (P1379, Sigma), followed by immunoblot to detect total mTOR (2972S, Cell Signaling Technology), p-mTOR (p-S2448, 2971S, Cell Signaling Technology), total p70S6K (2708S, Cell Signaling Technology), p-p70S6K (p-T421/S424, 9204S, Cell Signaling Technology), and Grb2 (610112, BD Biosciences), followed by fluorochrome conjugated secondary antibody binding (IRDye 800CW Goat anti-Rabbit [926-32211, LI-COR], IRDye 800CW Goat anti-Mouse [926-32210, LI-COR], IRDye 680RD Goat anti-Rabbit [926-68071, LI-COR], or IRDye 680RD Goat anti-Mouse [926-68070, LI-COR]). Images were obtained on the LI-COR Odyssey CLx and Image Studio 5.2 software platform.

### Statistics

Error bars in all figures represent the standard deviation (SD). Data were analyzed for statistical significance using GraphPad Prism. Indicators of statistical significance, and the test utilized, are found within the figure legends.

## Results

### L-Arginine Drives Anti-*Mtb* Macrophage Activity in the Presence and Absence of NO

It is well known that the amino acid L-arginine serves as the upstream metabolite for NO production by each of the three NO synthases (28). As such, the necessity of L-arginine during NO-mediated microbicidal macrophage activity *via* iNOS has been thoroughly documented (16, 29). Indeed, NO is critical for mouse host defense mechanisms against many mycobacterial species (7, 15, 17, 30–33). Still, the role of L-arginine in aiding macrophage anti-mycobacterial functions in the absence of NO production has received less attention. Therefore, we first examined the contribution of L-arginine during macrophage-mediated anti-*Mtb* immunity in the presence and absence of NO.

**Figure 1:**
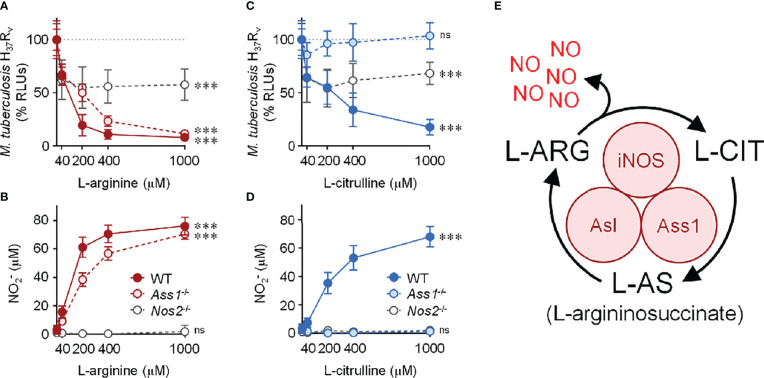
Contribution of L-arginine on anti-tuberculosis macrophage activity in the absence of nitric oxide. **(A–D)** WT (C57Bl/6 background), *Ass1* ^-/-^, or *Nos2* ^-/-^ PMΦs were infected with *Mtb* H_37_R_v_ (MOI~1) plus IFN-γ and cultured in R-free C-DMEM containing titrating amounts of L-arginine (red, A, B) or L-citrulline (blue, C, D). **(A, C)** RLUs were determined by measuring luminescence 72 hours post-infection and are normalized to 100% *Mtb* in macrophages without L-arginine or L-citrulline, represented by the dotted line. **(B, D)** Nitric oxide was evaluated by measuring nitrite (${\text{NO}}_{2}^{-}$) concentration by Griess (N ≥ 9, at least three experiments combined). ***p < 0.001 by 1-way analysis of variance (ANOVA) to determine an effect of L-arginine/L-citrulline on RLUs or${\text{ NO}}_{2}^{-}$ production; ns, not significant. Error bars, SD. **(E)** Schematic of nitric oxide (NO) production and L-arginine (L-ARG) biosynthesis from L-citrulline (L-CIT) in macrophages. iNOS, inducible nitric oxide synthase/nitric oxide synthase 2; Ass1, argininosuccinate synthase 1; Asl, argininosuccinate lyase.

Beginning with cell culture media devoid of L-arginine, we titrated L-arginine or L-citrulline to 40, 200, 400, or 1000 μM. Of note, common cell culture media contain L-arginine at 400 μM (DMEM) or 1000 μM (RPMI 1640). In IFN-γ-stimulated PMΦs, we observed a drop in *Mtb* as the concentration of L-arginine increased (**Figure 1A**). As expected, NO increased as macrophages were provided increasing concentrations of L-arginine (**Figure 1B**). A similar trend was observed when macrophages were provided L-citrulline in lieu of L-arginine, strengthening the contribution of L-arginine synthesis from L-citrulline for NO production and anti-mycobacterial function (**Figures 1C–E**). Yet, when macrophages were unable to synthesize L-arginine from L-citrulline by loss of either *Ass1* or *Asl*, they failed to reduce mycobacterial burden or produce NO when cultured in L-citrulline, even though they paralleled the phenotype of WT macrophages when exogenous L-arginine was provided (**Figure 1** and **S1A–D**). Importantly, when *Mtb* was cultured without macrophages, there was no effect of L-arginine or L-citrulline on *Mtb* growth, indicating the amino acids were acting on macrophages rather than directly inhibiting *Mtb* growth (**Figure S1E**). Analysis over time demonstrated that L-arginine or L-citrulline primarily resulted in bacteriostatic control of *Mtb* by macrophages (**Figure S1F**). Additionally, concentrations below 40 μM L-arginine or L-citrulline continued to display a dose-dependent reduction of *Mtb* burden (**Figures S1G, H**). Taken together, our data indicate that when extracellular L-arginine is unavailable, or when L-arginine synthesis is impaired, anti-mycobacterial macrophage function is compromised.

Considering the absolute necessity of L-arginine for NO production, we anticipated *Nos2* ^-/-^ macrophages – incapable in generating NO – would be unable to control *Mtb* infection. To our surprise, when cultured in L-arginine or L-citrulline, *Nos2* ^-/-^ macrophages were still able to reduce *Mtb* burden by nearly 50% (**Figure 1**). Together, our data suggest L-arginine is necessary not only for NO-mediated mycobacterial control, but also for anti-mycobacterial activity in the absence of NO.

### L-Arginine Propels Aerobic Glycolysis in *Mtb*-Infected Macrophages

**Figure 2:**
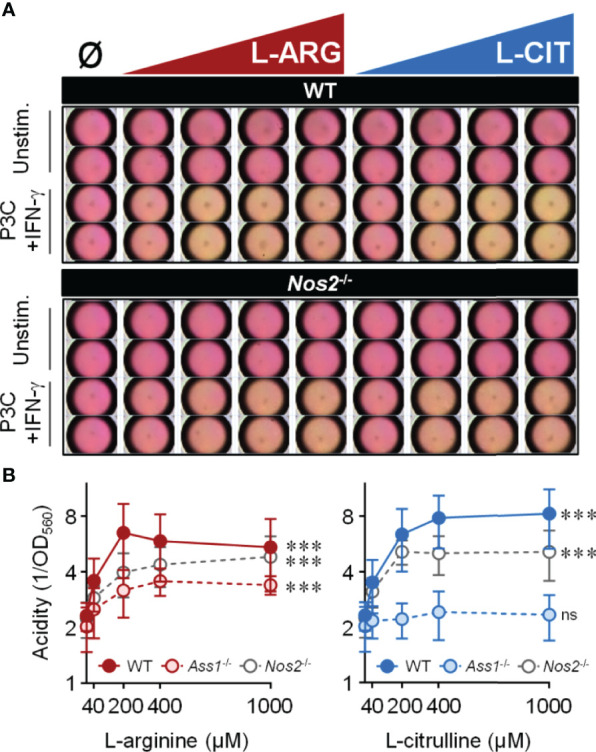
Extracellular acidity increases in the presence of L-arginine/L-citrulline. PMΦs were stimulated with Pam3Cys (P3C) plus IFN-γ cultured in R-free C-DMEM containing titrating amounts of L-arginine (red) or L-citrulline (blue). **(A)** Representative images of media color 72 hours post-stimulation. **(B)** Relative acidity from Pam3Cys plus IFN-γ stimulated PMΦs (N ≥ 9, at least three experiments combined). ***p < 0.001, by 1-way ANOVA to determine any effect of L-arginine/L-citrulline on acid production; ns, not significant. Error bars, SD.

*Mtb*-infected macrophages, or those stimulated with pathogen-associated molecular pattern (PAMP) molecules, display increased extracellular acidification associated with shifts in macrophage metabolic potential (34–37). We too noticed that cell culture media acidity (via a phenol red pH indicator) increased throughout infection. Surprisingly, we found that media acidity positively correlated with increasing L-arginine or L-citrulline concentrations. To eliminate the potential of *Mtb* directly causing an increase in acidity, we took a reductionist approach using the Toll-like receptor 2 (TLR2) agonist Pam3Cys with IFN-γ to stimulate macrophages. Following 72 hours, unstimulated macrophages maintained neutral pH (i.e. a consistent pink media color) regardless of amino acid concentration, indicating media pH was not directly altered by the biochemical properties of L-arginine or L-citrulline and these amino acids did not cause macrophage acid production in the absence of pro-inflammatory activation (**Figure 2A**). In IFN-γ and Pam3Cys stimulated WT or *Nos2* ^-/-^ macrophages, however, we observed an increase in acidity when either amino acid was present (**Figures 2A, B**). The increase in acid production following L-citrulline titration was dependent on L-arginine synthesis, since Ass1-deficient macrophages cultured in L-citrulline failed to generate acid (**Figure 2B**). L-arginine, L-lysine, and L-leucine have been reported to be detected by nutrient sensing pathways in order to regulate cellular activity (38–41). Yet, when macrophages lacked L-lysine or L-leucine, they still produced extracellular acid as long as L-arginine or L-citrulline were provided (**Figure S2A**), indicating that L-arginine is uniquely necessary for extracellular acidification in macrophages.

**Figure 3:**
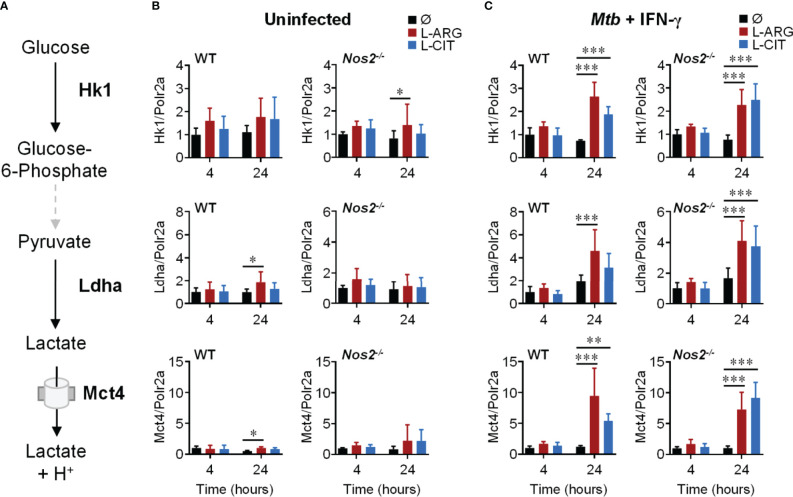
Transcriptional regulation of glycolysis genes by L-arginine/L-citrulline. **(A)** Schematic of glycolysis leading to lactate production and release from macrophages. **(B, C)** qRT-PCR analysis of uninfected PMΦs **(B)** or following *Mtb* H_37_R_v_ (MOI~1) infection and IFN-γ stimulation **(C)** from C57Bl/6 (WT) or *Nos2^-/-^* mice, cultured in R-free C-DMEM containing 400 μM L-arginine, L-citrulline, or neither amino acid (Ø). Gene expression is normalized to Polr2a. Data are the mean fold gene expression compared to 4 hour, neither amino acid data (N ≥ 5, two experiments combined). *p < 0.05, **p < 0.01, ***p < 0.001 by 2-way ANOVA with Sidak’s *post hoc* test. Error bars, SD.

Since extracellular acidification is an indicator of increased glycolysis, and given the importance of glycolysis in inflammatory macrophages (12, 42), we reasoned that L-arginine-mediated acid production was a result of increased glycolytic activity. We assessed the gene expression of integral mediators of glycolysis (**Figure 3A**), and observed the upregulation of Hk1, Ldha, and Mct4 in WT or *Nos2* ^-/-^ macrophages to be dependent on L-arginine and/or L-citrulline when infected with *Mtb* or stimulated with Pam3Cys plus IFN-γ (**Figures 3B, C** and **S2B**).

**Figure 4:**
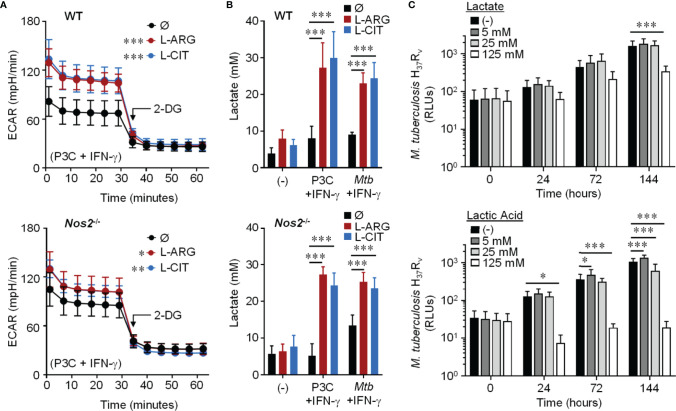
Effect of L-arginine/L-citrulline on macrophage glycolysis and lactate production. **(A)** PMΦs from C57Bl/6 (WT) or *Nos2^-/-^* mice were stimulated with Pam3Cys (P3C) plus IFN-γ in R-free C-DMEM containing 400 μM L-arginine, 400 μM L-citrulline, or neither amino acid for 24 hours. Following stimulation, cells were incubated in R-free Seahorse media containing 400 μM L-arginine (red), 400 μM L-citrulline (blue), or neither amino acid (black) for 1 hour prior to analysis *via* Seahorse XF technology. After 30 minutes, 2-DG (100 mM) was added to inhibit glycolysis. The extracellular acidification rate (ECAR) was determined over time (N ≥ 16, three experiments combined). **(B)** PMΦs from C57Bl/6 (WT) or *Nos2^-/-^* mice remained unstimulated (-) or were stimulated with P3C plus IFN-γ or infected with *Mtb* H_37_R_v_ (MOI~1) plus IFN-γ in R-free C-DMEM containing 400 μM L-arginine, L-citrulline, or neither amino acid for 72 hours. Lactate was measured from culture supernatants (N ≥ 6, at least two experiments combined). **(C)** *Mtb* H_37_R_v_ growth was determined following lactate or lactic acid addition by measuring luminescence at indicated time points. Data are the mean RLUs (N ≥ 12, at least two experiments combined). *p < 0.05, **p < 0.01, ***p < 0.001, by 1-way ANOVA to determine an effect of L-arginine/L-citrulline on ECAR **(A)**; by 2-way ANOVA with Tukey’s **(B)** or Dunnett’s **(C)** *post hoc* test. Error bars, SD.

We next examined the extracellular acidification rate (ECAR) following 24 hours of Pam3Cys and IFN-γ stimulation. When WT or *Nos2* ^-/-^ macrophages were provided L-arginine or L-citrulline, there was an increase in ECAR compared to macrophages cultured without either amino acid, which was ablated when the glycolysis inhibitor 2-deoxyglucose (2-DG) was added (**Figure 4A**). In line with this, we found a significant increase in secreted lactate from macrophages following Pam3Cys stimulation or *Mtb* infection with IFN-γ stimulation when L-arginine or L-citrulline were present compared to when neither were added to cell culture (**Figure 4B**). Importantly, neither lactate nor lactic acid were capable of directly reducing mycobacterial growth at physiological concentrations as *Mtb* growth was not inhibited until 5x the physiological concentration (125 mM) was added (**Figure 4C**). These data led us to speculate that glycolytic regulation by L-arginine serves as a mediator of macrophage host defense.

### mTOR Regulates L-Arginine-Mediated Glycolysis

**Figure 5:**
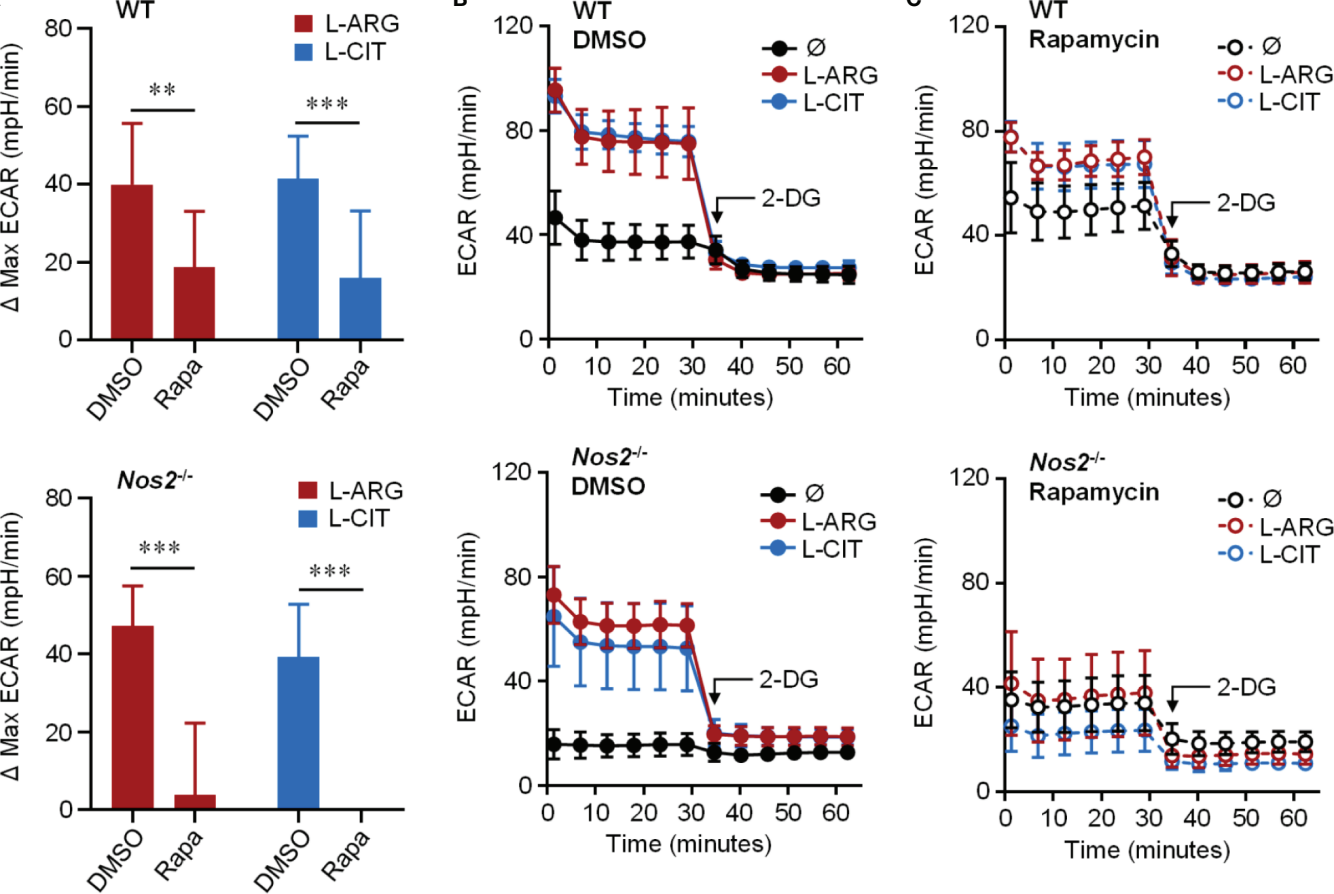
Requirement of mTORC1 for L-arginine/L-citrulline-enhanced glycolysis. PMΦs from C57Bl/6 (WT) or *Nos2^-/-^* mice were stimulated with Pam3Cys plus IFN-γ in R-free C-DMEM containing 400 μM L-arginine, L-citrulline, or neither amino acid with rapamycin or vehicle control (DMSO) for 24 hours. Following stimulation, cells were incubated in R-free Seahorse media containing 400 μM L-arginine (red), L-citrulline (blue), or neither amino acid (black) for 1 hour followed by ECAR analysis as in **Figure 4**. Data are shown as **(A)** the differences in maximum ECAR between DMSO and rapamycin groups or **(B, C)** the raw ECAR values (N = 10, WT, two experiments combined; N = 5, *Nos2* ^-/-^, one experiment). **p < 0.01, ***p < 0.001, by 2-way ANOVA with Sidak’s *post hoc* test. Error bars, SD.

Knowing that the mechanistic target of rapamycin complex 1 (mTORC1) is a common upstream regulator of glycolysis, and L-arginine is one of the few amino acids that activates mTORC1 (40), we hypothesized that L-arginine-mediated glycolysis relied on mTORC1 activity. When Pam3Cys and IFN-γ stimulated macrophages were provided the mTORC1 inhibitor rapamycin, there was a significant reduction in L-arginine/L-citrulline enhanced ECAR compared to those given DMSO vehicle control (**Figures 5A–C**). Similar findings were observed when macrophages lacked the ability to produce NO *via* genetic manipulation or chemical inhibition (**Figures 5A–C** and **S3A–C**). mTORC1 signaling *via* mTOR or p70S6K phosphorylation, however, was not driven by L-arginine (**Figure S3D**). Even though L-arginine failed to increase mTORC1 signaling, our data collectively indicate that mTOR regulates L-arginine-mediated glycolysis, regardless of the ability of the macrophage to produce NO.

### Glycolysis and mTOR Are Required for L-Arginine/L-Citrulline-Mediated Control of *Mtb* Burden in the Absence of NO

**Figure 6:**
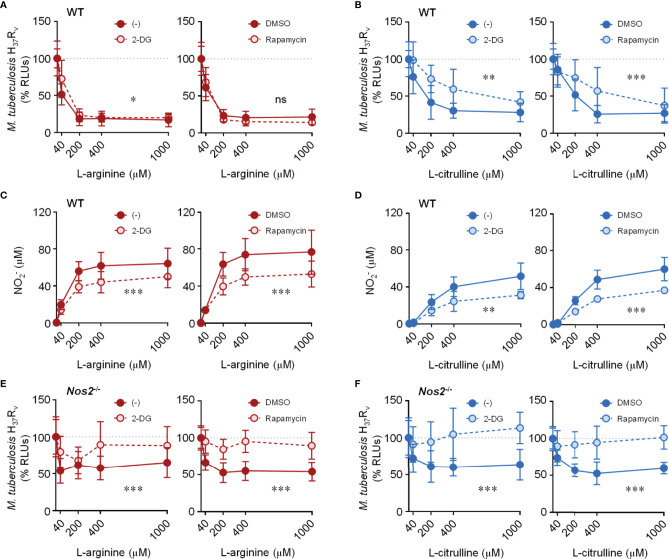
Effect of inhibiting glycolysis and mTORC1 activity on L-arginine/L-citrulline-mediated anti-mycobacterial function. PMΦs from C57Bl/6 (WT) or *Nos2^-/-^* mice were infected with *Mtb* H_37_R_v_ (MOI~1) plus IFN-γ cultured in R-free C-DMEM containing titrating amounts of L-arginine **(A, C, E)** or L-citrulline **(B, D, F)** with 2-DG (0.1 mM), rapamycin (20 nM), or appropriate controls. 72 hours post-infection *Mtb* RLUs **(A, B, E, F)** and ${\text{NO}}_{2}^{-}$ production **(C, D)** were determined as in **Figure 1** (N ≥ 6, at least two experiments combined). *p < 0.05, **p < 0.01, ***p < 0.001, by 2-way ANOVA to determine an effect between inhibitor and vehicle control; ns, not significant. Error bars, SD.

To address the necessity of glycolysis and/or mTORC1 on L-arginine/L-citrulline-mediated anti-mycobacterial macrophage activity, we turned to inhibiting these pathways in *Mtb*-infected macrophages. Optimal inhibitor concentrations were determined that reduced media acidity, but also had minimal effects on macrophage cytotoxicity (**Figure S4A, B**). We then cultured *Mtb* with these inhibitors found no effect on *Mtb* growth in the absence of macrophages (**Figure S4C**). Whether inhibiting glycolysis with 2-DG (0.1 mM), or mTOR with rapamycin (20 nM), we found WT macrophages to retain much of their L-arginine-mediated anti-mycobacterial activity (**Figures 6A, B**). Still, these chemicals only partially reduced NO production in WT macrophages (**Figures 6C, D**). In order to test the importance of glycolysis and mTOR signaling on L-arginine/L-citrulline mediated macrophage function in the absence of NO, we performed a similar experiment using *Nos2* ^-/-^ macrophages. Here, we found 2-DG and rapamycin were each able to inhibit L-arginine/L-citrulline-mediated anti-mycobacterial macrophage function (**Figures 6E, F**). Similar results were found when chemically inhibiting NO production with 1400w (**Figures S5A, B**). Finally, using torin (20 nM) in lieu of rapamycin to inhibit mTOR activity also blocked L-arginine/L-citrulline-mediated anti-mycobacterial macrophage function (**Figures S5C, D**).

Production of NO is largely driven by IFN-γ stimulation in infected macrophages. In the absence of IFN-γ costimulation where NO production was not detected, we found WT macrophages retained a significant amount of anti-*Mtb* activity mediated by L-arginine – similar to that observed in *Nos2* ^-/-^ macrophages co-stimulated with IFN-γ (**Figures S5E-J**). Still, the L-arginine-mediated anti-*Mtb* activity was lost in WT macrophages without IFN-γ when glycolysis or mTORC1 were inhibited (**Figures S5E, G, I**). Together, these data indicate that glycolysis and mTOR are necessary for regulating L-arginine-mediated macrophage control of *Mtb* burden in the absence of NO.

### L-Arginine-Mediated Control of *Mtb* Burden Is Associated With Enhanced Macrophage Number and the Necessity of this Amino Acid Cannot Be Surmounted by Exogenous NO

**Figure 7:**
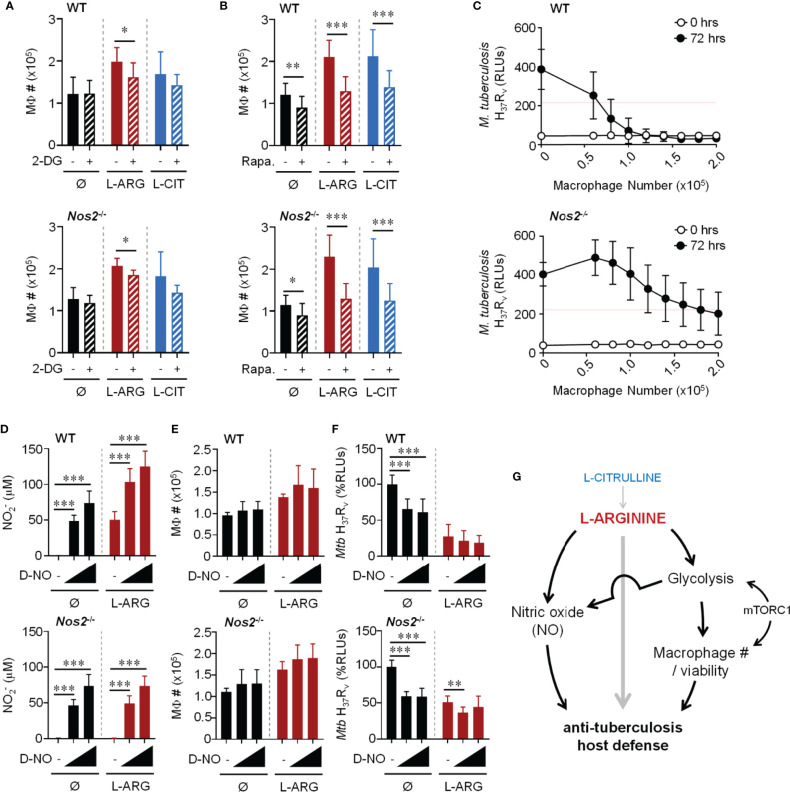
Dual role for L-arginine in driving anti-mycobacterial function *via* increasing macrophage number and NO production. PMΦs from C57Bl/6 (WT) or *Nos2^-/-^* mice were infected with *Mtb* H_37_R_v_ (MOI~1) plus IFN-γ cultured in R-free C-DMEM containing 400 μM L-arginine, L-citrulline, or neither amino acid (Ø) with 2-DG (0.1 mM) **(A)**, rapamycin (20 nM) **(B)**, or appropriate controls. Macrophage numbers were determined by crystal violet assay at 72 hours post-infection (N ≥ 6, at least two experiments combined). **(C)** Titrated numbers of PMΦs from WT or *Nos2* ^-/-^ mice were cultured with *Mtb* H_37_R_v_ (~2x10^5^ CFUs) plus IFN-γ in R-free C-DMEM containing 400 μM L-arginine. RLUs were determined immediately following infection and at 72 hours as in **Figure 1**. (N ≥ 6, at least 2 experiments combined, red line represents 50% of the change in *Mtb* RLUs between 0 and 72 hours in the absence of macrophages). **(D–F)** PMΦs from C57Bl/6 (WT) or *Nos2^-/-^* mice were infected with *Mtb* H_37_R_v_ (MOI~1) plus IFN-γ cultured in R-free C-DMEM containing 400 μM L-arginine or no L-arginine (Ø). DETA-NONOate (D-NO) was added at low (80 μM) or high (120 μM) concentrations indicated by the triangle, or not added (-). At 72 hours post-infection ${\text{NO}}_{2}^{-}$ production **(D)**, macrophage numbers **(E)**, and *Mtb* RLUs **(F)** and were determined as in **Figure 1** and above (N ≥ 6, at least two experiments combined). *p < 0.05, **p < 0.01, ***p < 0.001, by Student’s t test **(A, B)**; 1-way ANOVA with Fisher’s LSD *post hoc* analysis **(D–F)**. Error bars, SD. **(G)** Working model of pathways driven by L-arginine to assist in anti-mycobacterial macrophage function.

Bearing in mind the necessity of L-arginine to sustain cell viability, and even to promote proliferation (24, 43), we suspected L-arginine may regulate anti-*Mtb* activity in part by supporting macrophage numbers and viability. When *Mtb*-infected WT or *Nos2* ^-/-^ macrophages were cultured in the absence of L-arginine, we found fewer macrophages compared to cultures provided exogenous L-arginine or L-citrulline – a phenomenon only partially observed in uninfected macrophages (**Figures S6A, B**). Increases in macrophage numbers due to L-arginine or L-citrulline following infection were attenuated when inhibiting glycolysis or mTOR (**Figures 7A, B**). Similar findings were observed when analyzing macrophage viability 72 hours post-infection (**Figures S6C, D**). And even though uninfected macrophages were not as responsive to L-arginine/L-citrulline as those infected with *Mtb* concerning cell number, the viability of uninfected macrophages was increased when these amino acids were present in culture (**Figure S6E**). While macrophage proliferation was detected by 72 hours BrdU incorporation, it was minimal as compared to actively dividing RAW 264.7 macrophages cultured for a fraction of the time (**Figure S7**).

To determine the extent to which macrophage number contributed to host defense against *Mtb*, macrophages were titrated and cultured with ~2x10^5^ *Mtb* in 400 μM L-arginine (**Figure 7C**). All other experiments in this work started with 2x10^5^ macrophages with the same amount of *Mtb* for infection, which served here as the maximum number of macrophages in this particular experiment. Red lines on these graphs indicate 50% of the change in *Mtb* RLUs between 0 and 72 hours in the absence of macrophages. Compared to the number of WT macrophages needed to control *Mtb*, more than double *Nos2* ^-/-^ macrophages were required to control *Mtb* growth to the same degree (**Figure 7C**). This trend held true whether macrophages lacked NO due to genetic manipulation (*Nos2* ^-/-^), chemical inhibition (1400w), or lack of cytokine stimulation to trigger NO production (no IFN-γ) (**Figure S8**). It is not surprising that these data indicate a relationship between macrophage number and efficiency of combating *Mtb*. However, we additionally observe L-arginine to support macrophage number/viability during *Mtb* infection, and when compounded by the absence of NO, helps to shed light on how L-arginine contributes to host defenses independent of NO.

We next tested if exogenous NO could overcome the dependence on L-arginine for anti-mycobacterial function. We first found we could achieve similar concentrations of NO produced by WT macrophages by adding the NO donor, DETA-NONOate (D-NO) (**Figure 7D**). And while D-NO did not result in a loss of macrophage number or viability (**Figure 7E** amd **Figure S9A**), it was able to reduce *Mtb* RLUs when cultured with WT or *Nos2* ^-/-^ macrophages in the absence of L-arginine (**Figure 7F**). Interestingly, even in the absence of L-arginine, rapamycin blocked the D-NO effect on *Mtb* RLUs (**Figures S9B–I**), supporting the importance for mTOR in the absence of L-arginine in this model. Although D-NO resulted in similar concentrations of NO produced by WT macrophages, its addition was unable to reduce *Mtb* to amounts similar to those from cultures containing WT macrophages in L-arginine (**Figure 7F**), again supporting the concept that the contribution of L-arginine is greater than serving solely as a substrate for NO production during macrophage host defense. Given the above data, we propose a working model of L-arginine-mediated regulation of macrophage defenses against *Mtb*, with L-arginine directing NO-dependent and -independent avenues of anti-*Mtb* activity (**Figure 7G**).

## Discussion

Immunometabolism continues to emerge as a key contributor towards a comprehensive understanding of host-pathogen interactions. Our findings underscore the necessity of a single amino acid – L-arginine – in regulating aerobic glycolysis (i.e. Warburg effect), as well as macrophage number, viability, and subsequent host defense pathways. Low systemic L-arginine is observed in pulmonary tuberculosis patients compared to healthy controls (44). Furthermore, mycobacteria-mediated upregulation of the L-arginine consuming enzyme – arginase 1 (Arg1) – in macrophages decreases host defenses against this pathogen (45, 46). Additionally, L-arginine deprivation can suppress immunity against other infectious agents, including *Helicobacter pylori*, *Leishmania* spp., *Schistosoma mansoni*, and Hepatitis viruses (47–50). Given this, continuing to uncover how L-arginine and other immunonutrients regulate host metabolism is a significant area in immunology and infectious diseases research.

In the present study, we have proposed a model by which L-arginine and L-citrulline (following synthesis into L-arginine) aid in macrophage control of *Mtb* in the absence of NO production (**Figure 7G**). In mice, NO is critical in limiting mycobacterial infection, as *Nos2*-deficient mice are more susceptible to virulent *Mtb* infection and attenuated *M. bovis* bacille Calmette-Guérin (BCG) infection as compared to controls (17, 31). The role of NO in human macrophages during *Mtb* infection, however, is less clear. Both iNOS and NO have been detected in tissues from tuberculosis patients, and *NOS2* polymorphisms in humans are associated with increased susceptibility to *Mtb* (51–55). Still, human macrophages produce much less NO than mouse macrophages when infected *in vitro* (56, 57). NO amounts produced by human macrophages have been shown to play both a restrictive and beneficial role in *Mtb* growth, increasing the complexity of how and/or if physiologically relevant NO concentrations produced by human macrophages dictate *Mtb* viability (58, 59). Even with the differences between the contribution of human and mouse NO during infection – and perhaps because of them – continuing to investigate how L-arginine regulates macrophage function during infection independent of NO is warranted.

In addition to the role of NO on direct *Mtb* restriction, NO has been shown to have immunomodulatory effects itself. For example, the role of NO in modulating metabolic responses in mouse macrophages during lipopolysaccharide (LPS) stimulation has been well documented, including its connection to fracturing of the tricarboxylic acid cycle to promote inflammation (60–62). The global effect of inhibiting NO during infection with *Mtb* however demands further investigation, as *Mtb* infected macrophages are distinct from LPS-stimulated macrophages. In our model, we propose that inhibition of NO during infection with *Mtb* allows for otherwise masked host defense pathways to be revealed, as the effects of glycolysis and mTORC1 inhibition on *Mtb* control were only unveiled in NO-inhibited macrophages (**Figures 6** and **S5**). This suggests that NO may have suppressive effects on other host defense pathways. Indeed, NO has been linked to inhibition of NLRP3-dependent interleukin (IL)-1 responses and suppression of inflammatory lung pathology in *Mtb*-infected mice (63, 64). Still, additional experiments will be needed to examine *in vivo* how NO modulation in high and low L-arginine environments affects IL-1 and other cytokine signaling events, which might alter the inflammatory vigor of surrounding macrophages and other peripheral cells. We anticipate these and similar studies to highlight the importance of balancing anti-*Mtb* inflammation while refraining from excessive tissue damage, where L-arginine modulation has been described to play a central role (65).

Independent of the role L-arginine has on NO production, we found this amino acid to regulate macrophage number and viability (**Figures 7** and **S6**). Still, the number of viable macrophages needed to control *Mtb* burden was dependent on their ability to produce NO; more than double the number of macrophages were needed to control the same amount of *Mtb* when unable to produce NO as compared to those with sufficient NO production (**Figure 7C**). One can imagine the necessity of viable immune cells for efficient host defense mechanisms. For example, it has been shown that macrophage depletion prior to infection with *Yersinia pestis* results in higher blood and tissue colony forming units (CFUs) compared to mice with unaltered macrophage numbers (66). Additionally, macrophage depletion *via* clodronate has resulted in higher bacterial burden in the lungs after infection with *Mtb* (67). It is therefore not surprising that macrophage number and viability are necessary for efficient host defense in our experiments.

L-arginine was found to drive aerobic glycolysis in macrophages, and inhibiting glycolysis in the presence of L-arginine resulted in increased *Mtb* burden (**Figures 2** **–** **4**, **6**). Glycolysis has previously been shown to be positively correlated with L-arginine availability in heart tissue, tumor lines, T cells, and spermatozoa (68–71). Still, very little work has connected L-arginine in any fashion to glycolysis in macrophages. One report from Albina et al. has linked L-arginine to elevated glucose consumption and glycolysis in rat resident peritoneal macrophages (72). Increased glycolysis and glucose consumption associated with L-arginine, however, was dependent on macrophage NO production in their model (72). On the contrary, our research revealed L-arginine to drive glycolysis independent of macrophage NO production, which to our knowledge has not been previously described. Glycolysis is known to be important for macrophage responses to *Mtb*, as it is induced upon infection with *Mtb* and fuels many antimicrobial processes (12, 34, 73, 74). Several known anti-microbial mechanisms regulated by glycolysis include IL-1β production, NLRP3 inflammasome activation, and production of reactive oxygen/nitrogen species fueled by the pentose phosphate pathway (34, 75–77). Given our data demonstrating that L-arginine regulates glycolysis, the potential for L-arginine to regulate each of these anti-microbial processes is likely and requires further attention. Additionally, lung interstitial macrophages have been shown to increase glycolysis upon infection with *Mtb*, and that glycolysis is necessary for control of *Mtb in vivo* (67). These data complement work observing increased glycolysis following pulmonary mycobacterial infection *in vivo* and its requirement to control infection in mouse and human macrophages *in vitro* (34, 73, 74, 78, 79). Considering we find L-arginine to be upstream of glycolysis in macrophages, we are interested in exploring a link between L-arginine and glycolysis in other macrophage types including those from the lung. Furthermore, identifying how L-arginine might impact nodes of central carbon metabolism in addition to glycolysis, and how modulating L-arginine *in vivo* might alter macrophage metabolism and anti-tuberculosis host defense mechanisms, needs further attention.

Finally, we report that mTOR regulates L-arginine-driven macrophage glycolysis and *Mtb* control in the absence of NO (**Figures 5**, **6**). Although mTORC1 can be activated after sensing of L-arginine, L-leucine, and L-lysine (39–41), L-arginine (and L-citrulline) was the only amino acid to regulate acid production in stimulated macrophages (**Figure S2A**). Furthermore, L-arginine did not provide further mTORC1 activation beyond that induced following infection, suggesting that L-arginine was not directly working through mTOR to regulate glycolysis in *Mtb*-infected macrophages (**Figure S3D**). The role of mTOR during *Mtb* infection is likely multilayered. On one hand, a link has been established between mTORC1 and microbicidal autophagy in infected macrophages (80–83). For example, Gutierrez, et al. found that autophagy induced by inhibiting mTOR in macrophages led to decreased *M. bovis* BCG and *Mtb* survival and increased pathogen clearance (80). On the other hand, mTOR is recognized as a key mediator of glycolysis through Hypoxia Inducible Factor 1α (HIF-1α) activation (40, 79, 84, 85). Our present report parallels the latter, where a central role for mTOR in macrophages is to sustain anti-mycobacterial glycolytic activity. Certainly, experimental differences (e.g. timing, macrophage types, NO production, inoculation amount, etc.) could explain why inhibiting mTOR leads to decreased *Mtb* survival *via* autophagy in some reports, as well as increased *Mtb* burden in our model. Importantly, mTOR inhibition only led to increased *Mtb* when NO production was absent or reduced in our model, and may put our study in line with findings in human macrophages (86), which produce far less NO than those from mice. It is also possible that mTOR may have both a deleterious early role and a beneficial later role in pathogen clearance. The timing and context of altering mTOR activity would need to be carefully explored to determine when activating or inhibiting mTOR would be beneficial, rather than harmful, for host defense mechanisms.

Although mTOR regulates glycolysis through HIF-1α, we did not directly examine the role of this transcription factor. HIF-1α regulates IFN-γ-dependent glycolysis and subsequent control of *Mtb*, and works in concert with NO in mice to amplify aerobic glycolysis and suppress rampant inflammation *via* NFκB (73, 87). Additionally, HIF-1α regulates the conversion of pyruvate into lactate, which deprives *Mtb* of a preferred carbon source, ultimately leading to a reduction in *Mtb* growth (88). Given our data demonstrating that L-arginine regulates glycolysis and Ldha transcripts, it is intriguing to hypothesize that L-arginine regulates HIF-1α during *Mtb* infection. Furthermore, considering the increase in secreted lactate following infection, it would be worth investigating the impact lactate/lactic acid has on macrophages and/or other surrounding cells (e.g. T cells) integral to anti-mycobacterial host defenses – work we are currently pursuing. Regardless, the mechanism of how L-arginine regulates glycolysis remains to be elucidated.

A better understanding of metabolic pathways involved during infection is likely to provide insight and direction for the development of novel therapeutic strategies. Currently, targeting mTORC1 *via* rapamycin is used clinically to prevent organ transplant rejection, and has been shown to have anti-aging effects and anti-cancer properties (39). However, immunosuppression by rapamycin described here and by others may limit its benefits and could increase susceptibility to infections. Still, Lachmandas et al. showed previously that mice exposed to daily rapamycin treatment during the course of *Mtb* infection displayed decreased inflammatory cytokines from restimulated splenocytes, however CFU burden in control versus rapamycin treated mice remained unchanged (79). Therefore, further research into boosting the activity of mTORC1 or other downstream mediators during infection could provide insight into enhancing host immunity. Similarly, targeting glycolysis to upregulate macrophage metabolic activity during infection could provide benefits to host defense. Since glycolysis is regulated by L-arginine, a natural step would be to further examine amino acid metabolism and availability during infection. We have shown that throughout the course of infection with *M. bovis* BCG, serum L-arginine and L-citrulline amounts remained unchanged while lung concentrations were significantly altered, indicating that serum amino acid concentrations do not accurately reflect those at the site of infection (21). These data illustrate that further research into tissue specific amino acid availability would be beneficial to understanding fundamental cellular activation during infection. Current work in predicting tuberculosis onset and progression have included correlations of metabolite concentrations alone or in combination with transcriptomic data, and will be especially useful in identifying populations in tuberculosis endemic areas in which to focus therapeutic efforts (89, 90). Additional combinations of advanced metabolic analyses (e.g. MALDI-mass spectrometry imaging, [^18^F]-fluoro-D glucose/PET imaging) can be used to carefully evaluate metabolite availability and usage in infected tissues. These studies will ultimately lead to a more comprehensive understanding of the metabolic intricacies during infection, allowing for more precise drug discovery and development.

In summary, we propose an expanded model by which L-arginine regulates anti-mycobacterial host defense mechanisms in macrophages. Specifically, L-arginine and its precursor L-citrulline regulate glycolysis, which is regulated by mTOR, to enhance macrophage number, viability, and anti-mycobacterial responses in the absence of NO. Results here should be expanded to naïve, tissue-specific, and human macrophages to compare and contrast the effects of L-arginine/L-citrulline across a spectrum of macrophage types. And while our report demonstrated changes in macrophage number due to L-arginine/L-citrulline, future experiments aimed at analyzing if cell death and method(s) of cell death are altered by these amino acids are still needed. Furthermore, a more detailed analysis if/how L-arginine regulates the intracellular lifestyle of *Mtb* (inhibition of phagolysosome maturation, escape to cytosol, balance of necrosis vs. apoptosis, etc.) is warranted. Additional studies analyzing specific metabolic signatures and metabolite availability at the site of infection will be crucial to understanding host-pathogen interactions during tuberculosis and other infectious diseases. Collectively, we expect that a more complete understanding of immunometabolism will provide the knowledge needed to accurately target host immunity in response to invading pathogens.

## Data Availability Statement

The raw data supporting the conclusions of this article will be made available by the authors, without undue reservation.

## Ethics Statement

The animal study was reviewed and approved by Cincinnati Children’s Hospital Medical Center IACUC.

## Author Contributions

Conceptualization, MM and JQ. Methodology, all authors. Investigation, MM, RRC, SS, and JQ. Resources, EJ and JQ. Writing – Original Draft, MM and JQ. Writing – Review and Editing, all authors. Supervision, EJ and JQ. Funding Acquisition, MM and JQ. All authors contributed to the article and approved the submitted version.

## Funding

This work was supported by the National Institutes of Health R01AI116668 (JQ) and T32AI118697 (MM appointee), the American Lung Association IA-696872 (JQ), a Research Innovation and Pilot Award from Cincinnati Children’s Hospital Medical Center (JQ), the Albert J. Ryan Fellowship (MM), the University of Cincinnati Graduate Student Government Research Fellowship (MM), and the Division of Infectious Diseases at Cincinnati Children’s Hospital Medical Center.

## Disclaimer

The content is solely the responsibility of the authors and does not necessarily represent the views of the funding sources.

## Conflict of Interest

The authors declare that the research was conducted in the absence of any commercial or financial relationships that could be construed as a potential conflict of interest.

## Abbreviations

2-DG, 2-deoxyglucose; ANOVA, Analysis of Variance; Arg1, Arginase 1; Asl, Argininosuccinate Lyase; Ass1, Argininosuccinate Synthase 1; BCG, Bacille Calmette-Guérin; BrdU, 5-bromo-2’-deoxyuridine; CCHMC, Cincinnati Children’s Hospital Medical Center; CFUs, Colony Forming Units; DMEM, Dulbecco’s Modified Eagles Media; DMSO, Dimethyl Sulfoxide; D-NO, DETA-NONOate; ECAR, Extracellular Acidification Rate; HIF-1α, Hypoxia Inducible Factor 1α; Hk1, Hexokinase 1; IFN-γ, Interferon-γ; IL-1, Interleukin 1; iNOS, Inducible Nitric Oxide Synthase; L-ARG, L-Arginine; L-CIT, L-Citrulline; Ldha, Lactate Dehydrogenase A; LPS, Lipopolysaccharide; Mct4, Monocarboxylate Transporter 4; MOI, Multiplicity of Infection; *Mtb*, *Mycobacterium tuberculosis*; mTOR, Mechanistic Target of Rapamycin; mTORC1, Mechanistic Target of Rapamycin Complex 1; MΦ, Macrophage; NO, Nitric Oxide; PAMP, Pathogen Associated Molecular Pattern; PBS, Phosphate Buffered Saline; PMΦs, Peritoneal Macrophages; Polr2a, RNA Polymerase 2 Subunit A; qRT-PCR, Quantitative Real Time Polymerase Chain Reaction; Rapa, Rapamycin; R-free, L-Arginine-Free; RFU, Relative Fluorescence Units; RLUs, Relative Luminescence Units; RPMI, Roswell Park Memorial Institute; SD, Standard Deviation; TLR, Toll-Like Receptor; WT, Wild Type.
