# Supplementary material for: Promotion of Anti-Tuberculosis Macrophage Activity by L-Arginine in the Absence of Nitric Oxide
Source: Front Immunol. 2021 May 14;12:653571. doi: 10.3389/fimmu.2021.653571 (PMC8160513; doi:10.3389/fimmu.2021.653571)
Supplement: Supplementary file 1 [file DataSheet_1.pdf]

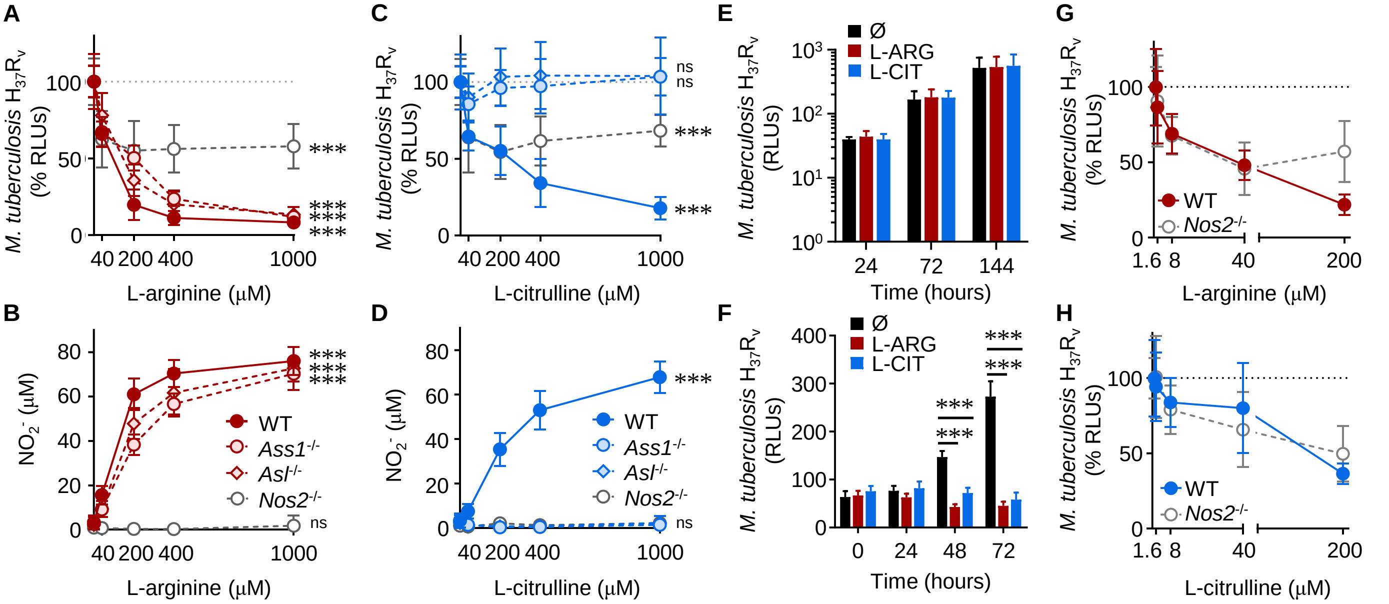

Supplemental Figure 1. Correlation of available L-arginine/L-citrulline with *M. tuberculosis* viability in macrophages. (A-D, G, H) *Nos2*$^{-/-}$, *Assl*$^{-/-}$, *Asl*$^{-/-}$ or WT (C57Bl/6 background) PMΦs were infected with *Mtb* $H_{37}R_v$ (MOI~1) plus IFN-γ. RLUs and NO_2^- were determined at 72 hours post-infection as described in Figure 1 (N ≥ 9, at least three experiments combined (A-D); N ≥ 5, two experiments combined (G, H)). (E, F) *Mtb* $H_{37}R_v$ was cultured in 400 μM L-arginine, L-citrulline, or neither amino acid in the absence (E) or presence (F) of C57Bl/6 PMΦs. Luminescence was measured at indicated times (N = 6, combined from two experiments (E); representative of three experiments (F)). ***p < 0.001 by 1-way ANOVA to determine an effect of L-arginine/L-citrulline on RLUs or NO_2^- production (A-D); by 2-way ANOVA with Dunnett's *post hoc* analysis (F); ns, not significant. Error bars, SD. (A-D) Data from WT, *Ass1*$^{-/-}$, and *Nos2*$^{-/-}$ are duplicated from Figure 1.

Supplemental Figure 2

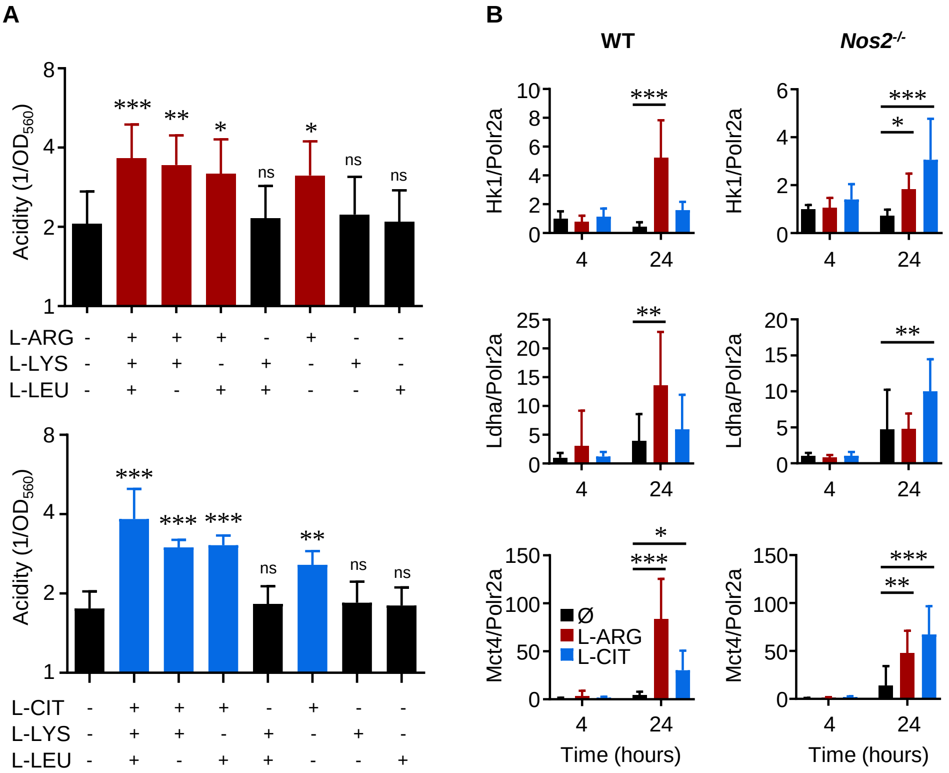

Supplemental Figure 2. L-arginine/L-citrulline-mediated regulation of macrophage acid production and glycolysis transcripts. (A) Acid production was determined as in Figure 2 from Pam3Cys and IFN-γ stimulated PMΦs with and without 400 µM L-arginine/L-citrulline, 800 µM L-lysine, or 400 µM L-leucine as indicated (N ≥ 9, at least 3 experiments combined). (B) qRT-PCR analysis following 4 and 24 hours Pam3Cys and IFN-γ stimulation of PMΦs from C57Bl/6 (WT) or *Nos2*$^{-/-}$ mice, cultured in R-free C-DMEM containing 400 µM L-arginine, L-citrulline, or neither amino acid (Ø) (N ≥ 6, two experiments combined). Gene expression data are normalized to Polr2a and are the mean fold gene expression compared to 4 hour, neither amino acid. *$p < 0.05$, **$p < 0.01$, ***$p < 0.001$, by 1-way ANOVA with Dunnett's *post hoc* analysis comparing indicated acidity to that from PDMs lacking L-arginine/L-citrulline, L-lysine, and L-leucine (A); 2-way ANOVA with Sidak's *post hoc* analysis (B). Error bars, SD.

Supplemental Figure 3

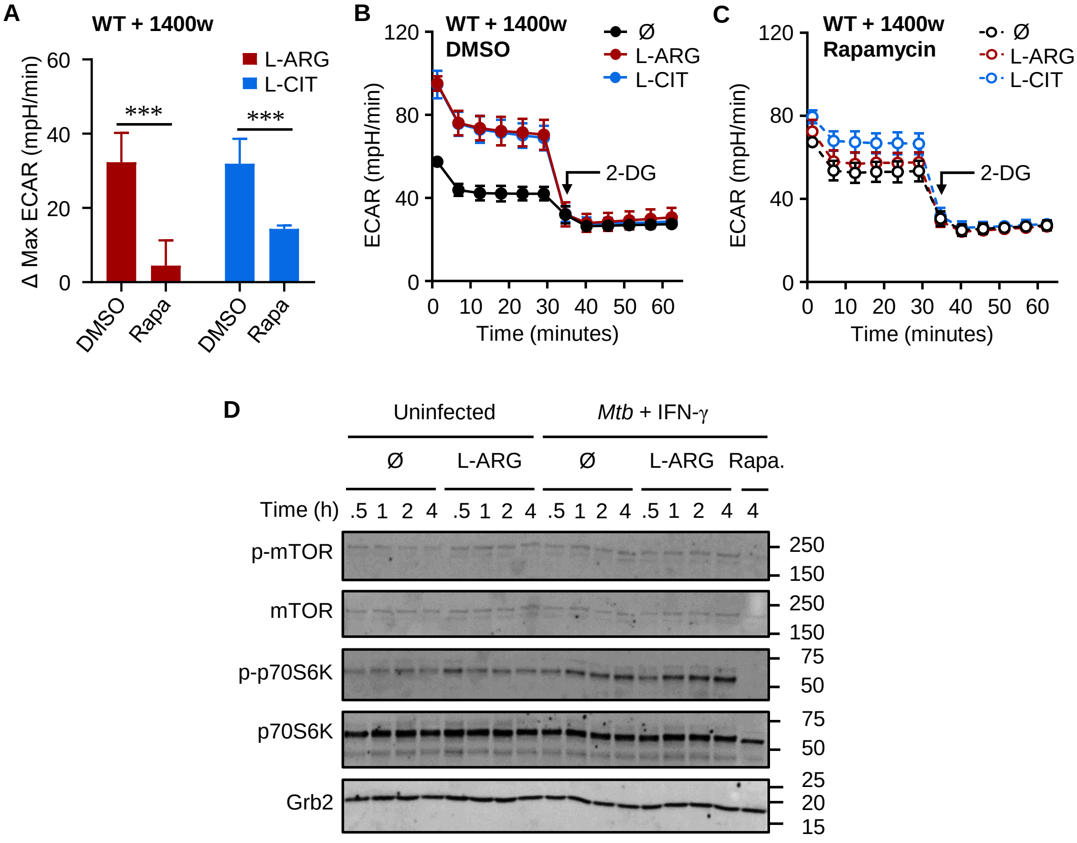

Supplemental Figure 3. Requirement of mTORC1 for L-arginine/L-citrulline-enhanced glycolysis. PMΦs from C57Bl/6 (WT) mice were treated with 1400w and stimulated with Pam3Cys plus IFN-γ in R-free C-DMEM containing 400 μM L-arginine, L-citrulline, or neither amino acid with rapamycin or vehicle control (DMSO) for 24 hours. Following stimulation, cells were incubated in R-free Seahorse media containing 400 μM L-arginine (red), L-citrulline (blue), or neither amino acid (black) for 1 hour, and ECAR was determined as in Figure 3. Data are shown as (A) the difference in maximum ECAR between DMSO and rapamycin groups, or (B, C) the raw ECAR values (N = 5, one experiment). (D) C57Bl/6 PMΦs were infected with *Mtb* $H_{37}R_a$ (MOI~1) plus IFN-γ in R-free C-DMEM with or without (Ø) 400 μM L-arginine. Protein lysates were collected at the indicated times and analyzed by immunoblot. (representative of two experiments). ***p < 0.001, by 2-way ANOVA with Sidak's *post hoc* test (A). Error bars, SD.

Supplemental Figure 4

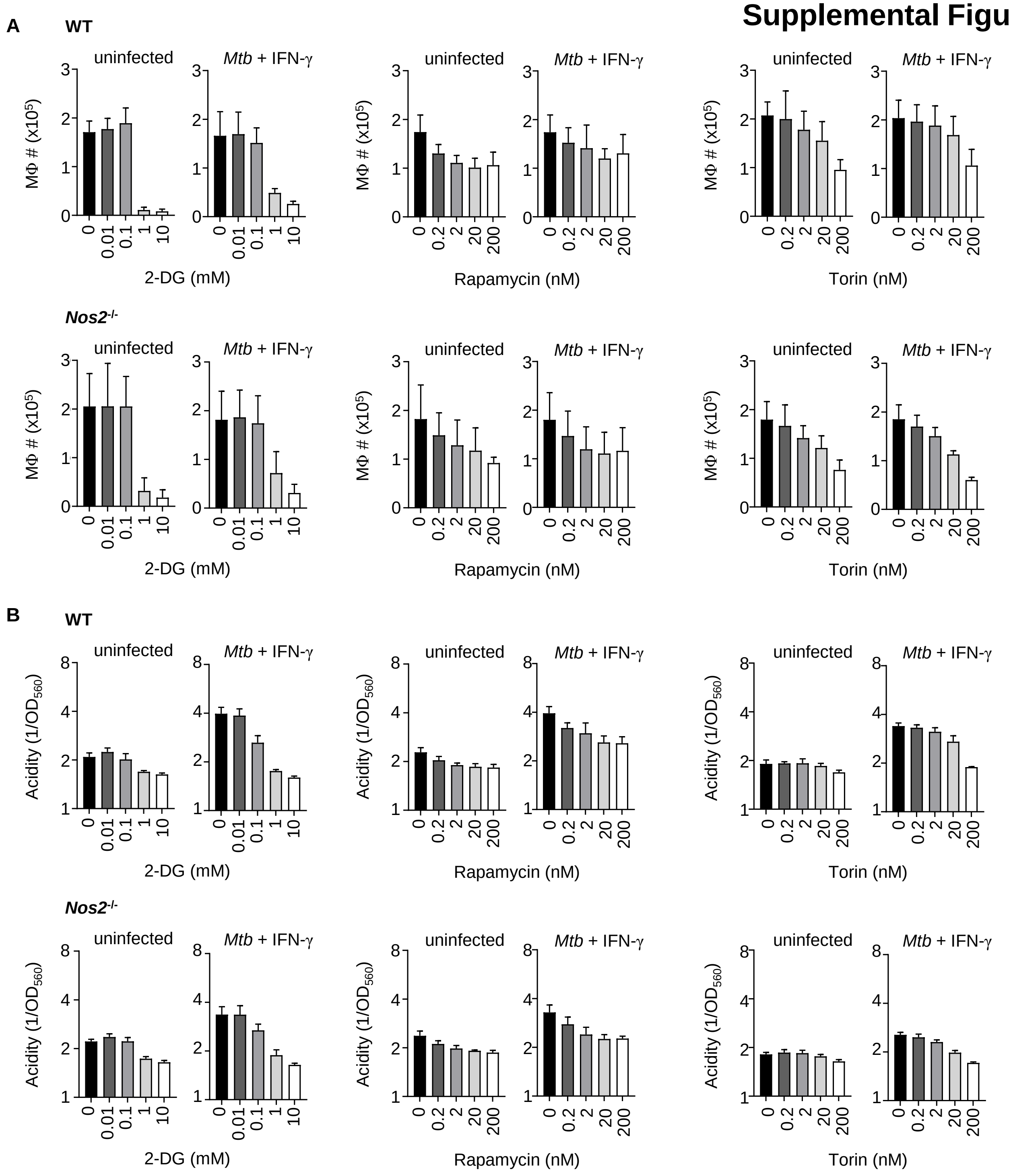

Supplemental Figure 4. Inhibitor titration. (A, B) PMΦs from C57Bl/6 (WT) and *Nos2*$^{-/-}$ mice were infected with *Mtb* $H_{37}R_a$ plus IFN-γ or remained uninfected in R-free C-DMEM containing 400 μM L-arginine with 2-DG (N ≥ 9), rapamycin (N ≥ 6), torin (N ≥ 3), or vehicle control for 72 hours (combined from or representative of three experiments). Cultures were then analyzed for macrophage number by crystal violet staining (A) or media acidity (B). (C) RLUs from *Mtb* $H_{37}R_v$ cultured without macrophages in 400 μM L-arginine containing C-DMEM plus 2-DG (0.1 mM), rapamycin (20 nM), torin (20 nM) or appropriate controls (N ≥ 12, two experiments combined). Error bars, SD.

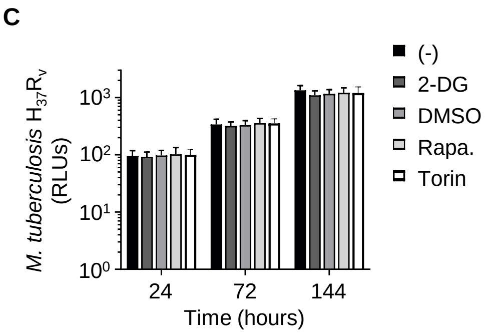

Supplemental Figure 5

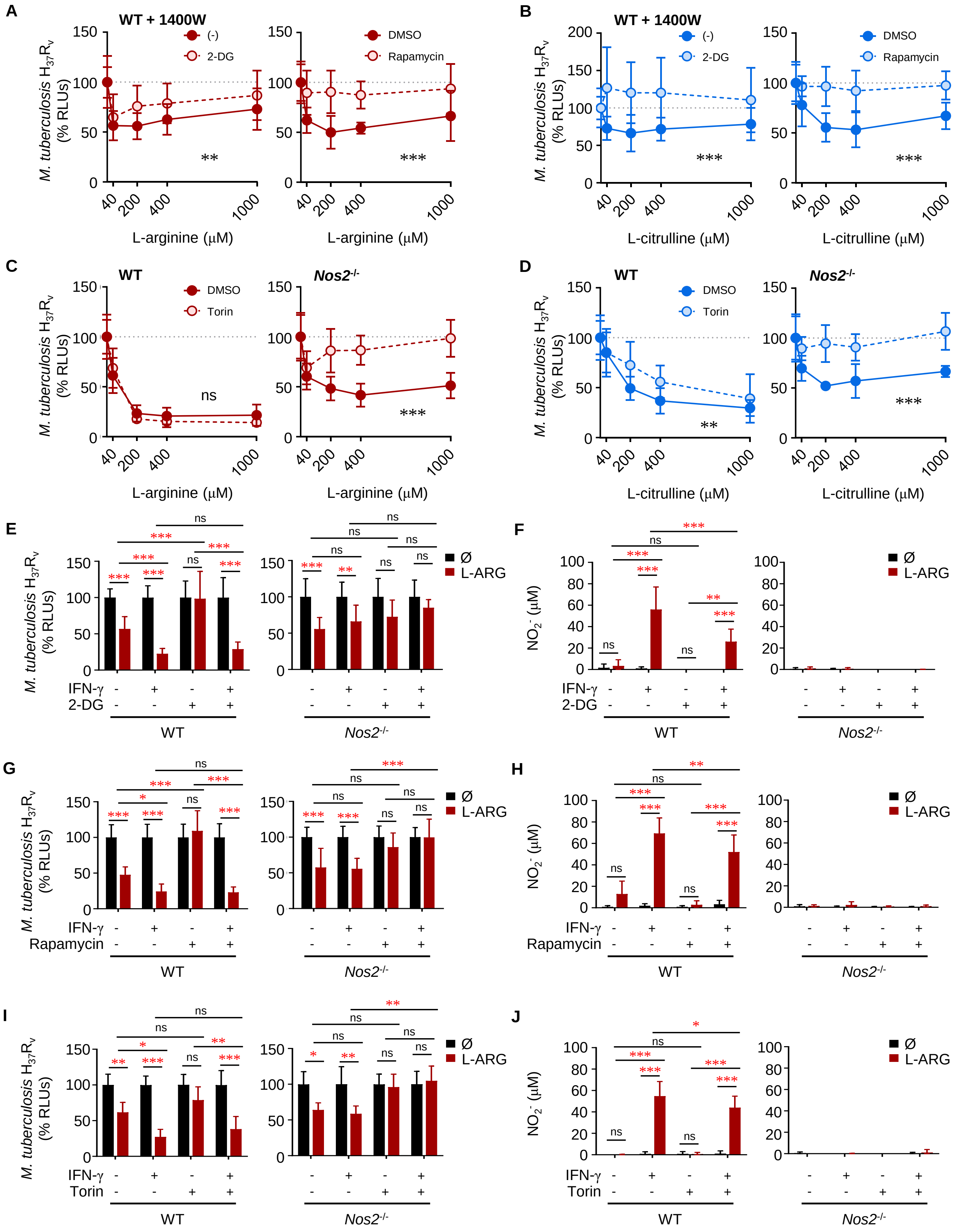

Supplemental Figure 5. Effect of inhibiting glycolysis or mTORC1 activity on L-arginine/L-citrulline-mediated anti-mycobacterial function. (A-D) PMΦs from C57Bl/6 (WT) or *Nos2*$^{-/-}$ mice were infected with *Mtb* $H_{37}R_v$ (MOI~1) plus IFN-γ in R-free C-DMEM containing titrating amounts of L-arginine (A, C) or L-citrulline (B, D) with 1400w plus 2-DG (0.1 mM) or rapamycin (20 nM) (A, B), or torin (20 nM) (C, D) with appropriate controls. 72 hours post-infection *Mtb* RLUs were determined as in Figure 1 ($N \geq 6$, at least two experiments combined). (E-J) PMΦs from C57Bl/6 (WT) or *Nos2*$^{-/-}$ mice were infected with *Mtb* $H_{37}R_v$ with or without IFN-γ in R-free C-DMEM containing 400 μM L-arginine or no L-arginine (Ø) with 2-DG (0.1 mM) (E, F), rapamycin (20 nM) (G, H), or torin (20 nM) (I, J). *Mtb* RLUs and NO_2^- production were determined 72 hours post-infection as in Figure 1 ($N \geq 6$, at least two experiments combined). RLUs from cultures without L-arginine were normalized to 100% for each treatment group. *$p < 0.05$, **$p < 0.01$, ***$p < 0.001$, by 2-way ANOVA to determine an effect between inhibitor and vehicle control (A-D); 2-way ANOVA with Sidak's *post hoc* analysis (E-J); ns, not significant. Error bars, SD.

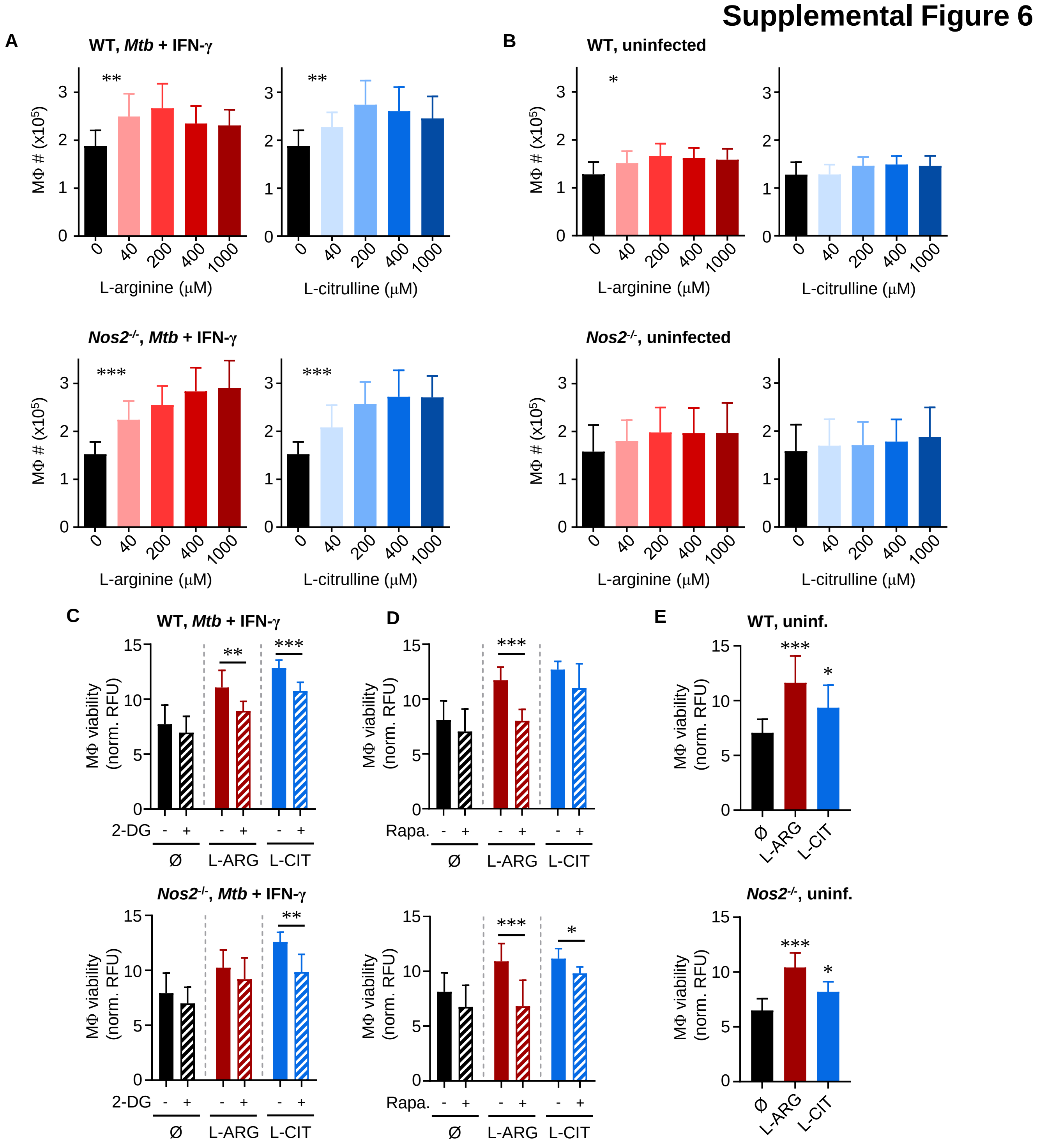

Supplemental Figure 6. Effect of L-arginine/L-citrulline on macrophage number and viability. (A, B) PMΦs from C57Bl/6 (WT) or *Nos2*$^{-/-}$ mice were infected with *Mtb* $H_{37}R_v$ (MOI~1) plus IFN-γ (A) or remained uninfected (B) in R-free C-DMEM containing titrating amounts of L-arginine (red) or L-citrulline (blue). Macrophage numbers were determined by crystal violet assay 72 hours post-infection (N ≥ 6, at least two experiments combined). (C-E) PMΦs from C57Bl/6 (WT) or *Nos2*$^{-/-}$ mice were infected with *Mtb* $H_{37}R_v$ (MOI~1) plus IFN-γ (C, D) or remained uninfected (E) in R-free C-DMEM containing 400 μM L-arginine, L-citrulline, or neither amino acid (Ø) with 2-DG (0.1 mM) (C), rapamycin (20 nM) (D), or appropriate controls. Macrophage vitality was determined by alamarBlue assay at 72 hours post-infection (N ≥ 6, at least two experiments combined). *p < 0.05, **p < 0.01, ***p < 0.001, by 1-way ANOVA (A, B); Student's t test (C, D); 1-way ANOVA with Dunnett's *post hoc* analysis (E) comparing the indicated column to those without amino acids. Error bars, SD.

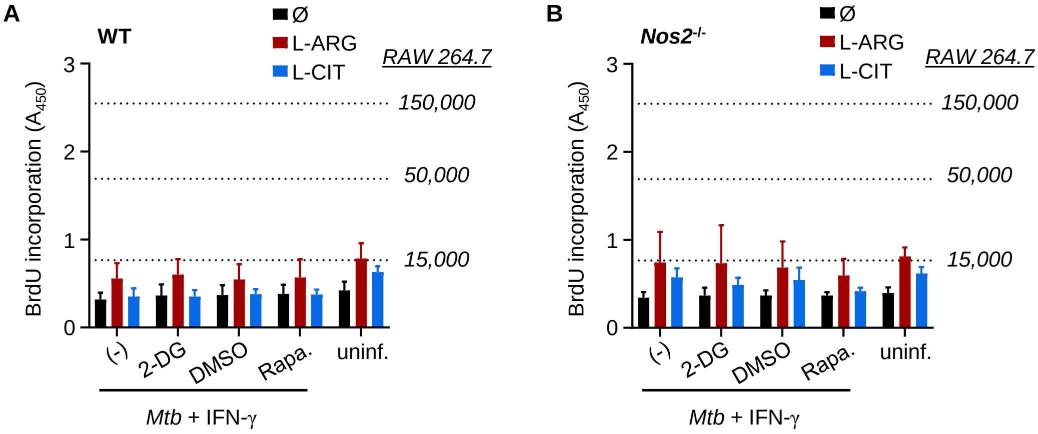

Supplemental Figure 7. Effect of L-arginine/L-citrulline on macrophage proliferation. PMΦs from C57Bl/6 (WT) (A) or *Nos2*$^{-/-}$ (B) mice were infected with *Mtb* $H_{37}R_v$ (MOI~1) plus IFN-γ or remained uninfected in R-free C-DMEM containing 400 μM L-arginine, L-citrulline, or neither amino acid (Ø) with 2-DG (0.1 mM), rapamycin (20 nM), or appropriate controls. BrdU was added immediately following infection to allow for 72 hours nucleotide incorporation. RAW 264.7 macrophages were cultured at 15, 50, and 150 x 10^3 cells/well in C-RPMI with BrdU for 20 hours as a positive control for proliferation (as indicated by the lines). Macrophage proliferation was determined at 72 hours post-infection (PMΦs) or 20 hours post plating (RAW 264.7) by BrdU assay (*Mtb* + IFN-γ, N ≥ 6, at least two experiments combined; uninfected, N = 3, one representative of two experiments). Error bars, SD.

Supplemental Figure 8

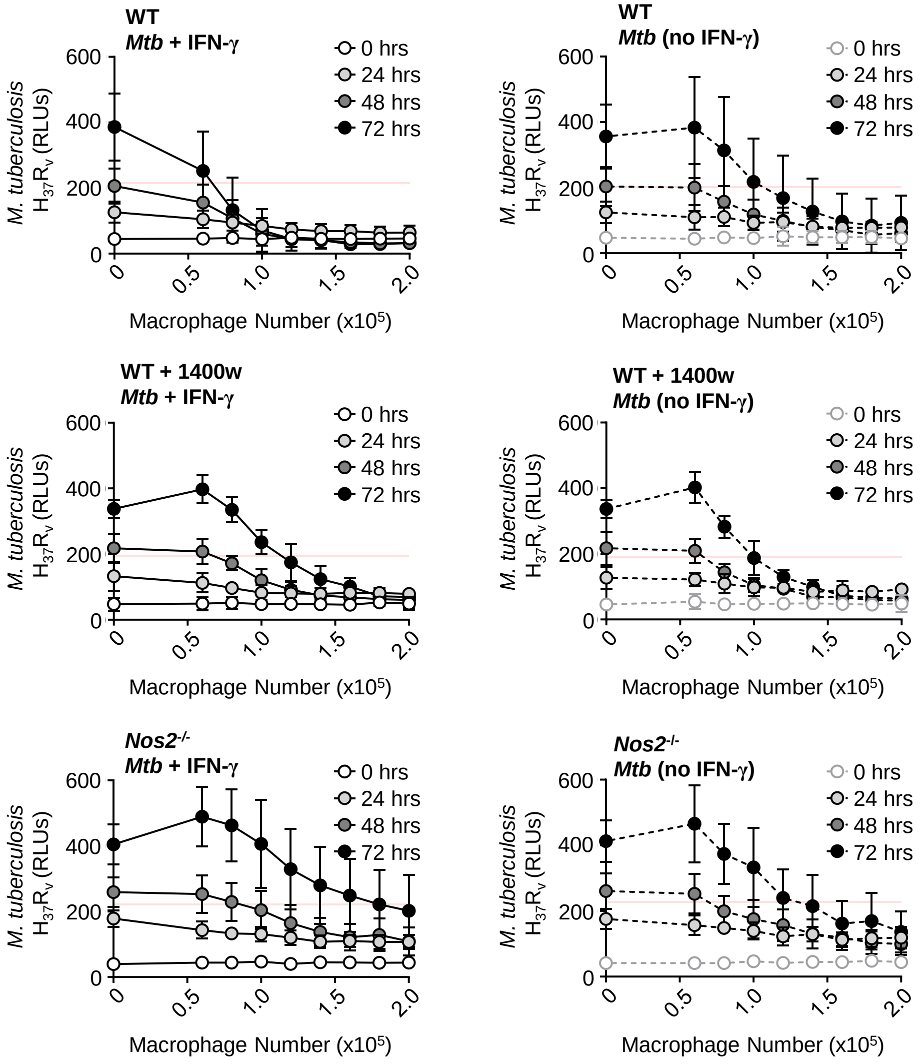

Supplemental Figure 8. Impact of macrophage number on *Mtb* viability in the presence / absence of IFN-γ and NO production. Titrated numbers of PMΦs from WT or *Nos2*$^{-/-}$ mice were infected with *Mtb* $H_{37}R_v$ (~2x10^5 CFUs) in R-free C-DMEM containing 400 μM L-arginine with and without IFN-γ as indicated. WT PDMs were also treated with 1400W to chemically inhibit NO production as indicated. RLUs were determined immediately following addition of mycobacteria and at 24, 48, and 72 hours post-infection. (N ≥ 6, at least 2 experiments combined, red line represents 50% of the change in *Mtb* RLUs between 0 and 72 hours in the absence of macrophages). Error bars, SD. Data from WT and *Nos2*$^{-/-}$ PMΦs with *Mtb* plus IFN-γ at 0 and 72 hours are duplicated from Figure 7.

Supplemental Figure 9

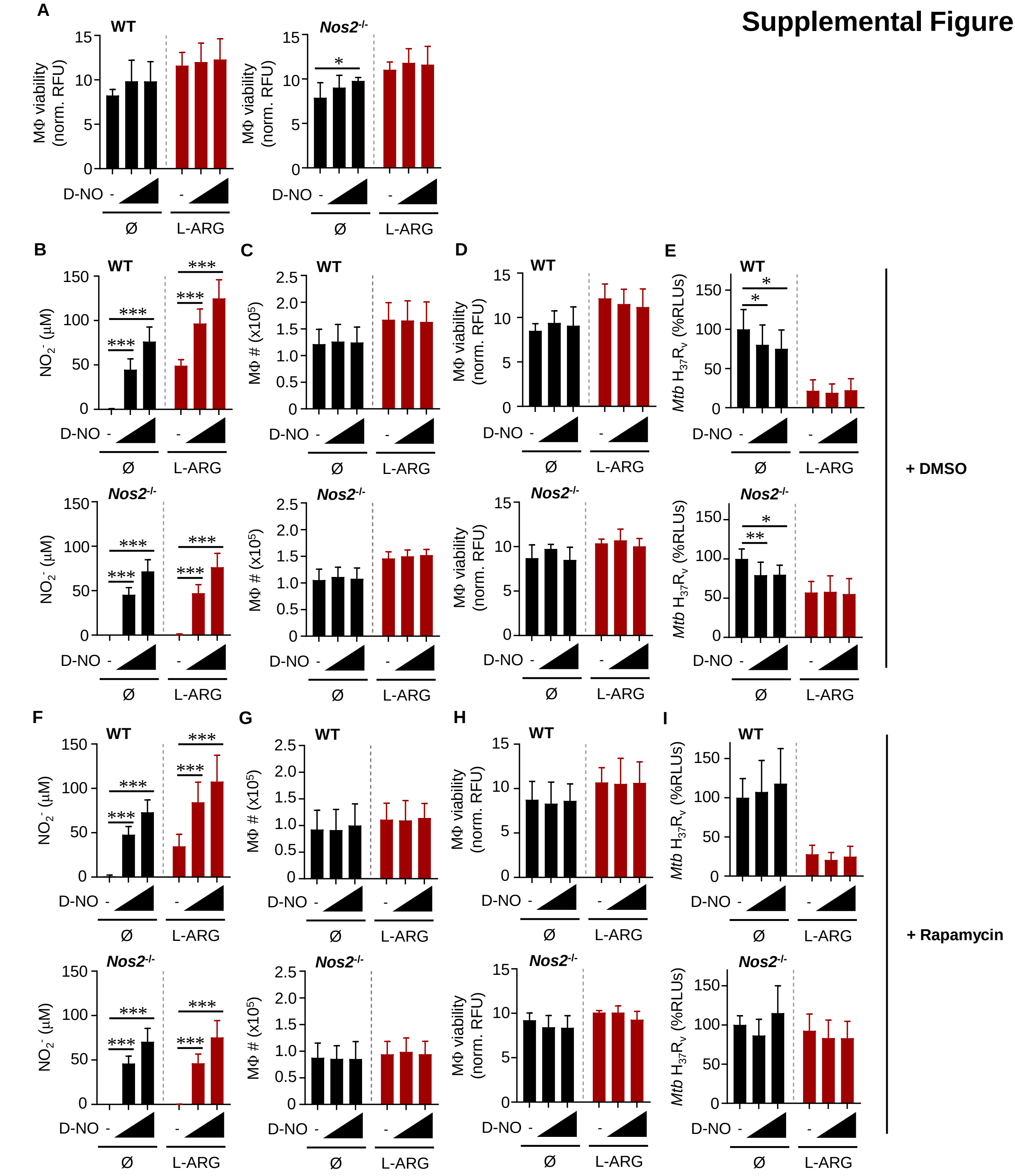

Supplemental Figure 9. Effect of exogenous NO on macrophage number, viability, and anti-mycobacterial activity. PMΦs from C57Bl/6 (WT) or *Nos2*$^{-/-}$ mice were infected with *Mtb* $H_{37}R_v$ (MOI~1) plus IFN-γ cultured in R-free C-DMEM containing 400 μM L-arginine or no L-arginine (Ø) without further treatment (A), or with rapamycin (20 nM) or vehicle control (DMSO) (B-I) . DETA-NONOate (D-NO) was added at low (80 μM) or high (120 μM) concentrations indicated by the triangle, or not added (-). At 72 hours post-infection macrophage viability (A, D, H) NO_2^- production (B, F), macrophage numbers (C, G), and *Mtb* RLUs (E, I) and were determined as in Figures 1 and 7 (N ≥ 6, at least two experiments combined). *p < 0.05, **p < 0.01, ***p < 0.001, by 1-way ANOVA with Fisher's LSD *post hoc* analysis. Error bars, SD.
